# Exercise, manipulation and traction physiotherapy in the conservative management of lumbar disc herniation: A systematic review and meta-analysis

**DOI:** 10.1016/j.bas.2025.105632

**Published:** 2025-10-16

**Authors:** Santhosh G. Thavarajasingam, Daniele S.C. Ramsay, Srikar R. Namireddy, Abith G. Kamath, Sree Kanakala, Hasan Zaidi, Rishi Parikh, Amaan Peerbhai, Hariharan Subbiah Ponniah, Aksaan Arif, Ahmed Salih, Ahkash Thavarajasingam, Jonathan Neuhoff, Daniel Scurtu, Dragan Jankovic, Andreas Kramer, Florian Ringel

**Affiliations:** a Imperial Brain & Spine Initiative, Imperial College London, London, United Kingdom; b Faculty of Medicine, Imperial College London, London, United Kingdom; c Faculty of Medicine, Medizinische Hochschule Hannover, Hannover, Germany; d Center for Spinal Surgery and Neurotraumatology, Berufsgenossenschaftliche Unfallklinik Frankfurt am Main, Germany; e Department of Neurosurgery, LMU University Hospital, LMU Munich, Munich, Germany; f EANS Spine Section, European Association of Neurosurgical Societies, Brussels, Belgium

**Keywords:** Lumbar disc herniation, Physiotherapy, Exercise therapy, Manipulation therapy, Traction therapy, Conservative treatment, Systematic review, Meta-analysis

## Abstract

**Introduction:**

Lumbar disc herniation (LDH) is a leading cause of global back pain with significant socioeconomic impact. Conservative physiotherapy, including exercise, manipulation, and traction therapies, is a common first-line treatment. However, their relative efficacy and applicability to specific subgroups remain unclear.

**Research question:**

This systematic review and meta-analysis evaluated the efficacy of these three modalities, identified factors influencing variability, and explored subgroup-specific applications.

**Material and methods:**

Following PRISMA guidelines, a systematic review was conducted with searches of PubMed, MEDLINE, EMBASE, OVID, Scopus, and grey literature. Forty-three studies were included in the qualitative synthesis and 20 in the meta-analysis. Random-effects models estimated pooled standardized mean changes (SMCs), and meta-regression examined covariates influencing variability.

**Results:**

The pooled SMC across modalities was 2.28 (95 % CI: 1.51, 3.05), indicating large treatment effects, though heterogeneity was high (I^2^ = 97.9 %). Traction therapy had the highest effect size (SMC = 2.52, 95 % CI: 1.57, 3.37), followed by exercise therapy (SMC = 1.97, 95 % CI: 0.46, 3.48) and manipulation therapy (SMC = 1.91, 95 % CI: 0.24, 4.04). Follow-up duration significantly influenced effect sizes (p < 0.001), with shorter durations associated with larger effects. Qualitative findings suggested potential subgroup benefits for complex or chronic pain patients, but quantitative evidence for subgroup differentiation was limited.

**Discussion and conclusion:**

Conservative therapies may effectively reduce LDH-related pain and disability, with traction therapy demonstrating the largest pooled effect size. High heterogeneity and methodological inconsistencies limit subgroup-specific recommendations. Rigorous trials and standardized methodologies are essential for optimizing evidence-based care for LDH patients.

## Highlights

• Traction therapy demonstrated the largest effect size among modalities (SMC = 2.52, 95 % CI: 1.57–3.37).
• High heterogeneity (I^2^ = 97.9 %) highlights variability in protocols, populations, and follow-up durations.
• Follow-up duration significantly influenced outcomes, with shorter durations showing exaggerated effects (p < 0.001).
• Despite strong effects, methodological inconsistencies limit subgroup-specific therapy recommendations.
• Findings emphasize the need for standardized protocols and head-to-head comparisons to guide personalized LDH care.

## 1 Introduction

Back pain is one of the most common presenting complaints globally, with lumbar disc herniation (LDH) being the most common differential diagnosis (Wong et al., 2023). Back pain is estimated to cost the USA between 100 and 200 billion dollars (about $620 per person in the US) annually (Katz, 2006; Al Qaraghli and De Jesus, 2025). European studies indicate that the annual incidence of herniated disc ranges from 5 to 20 cases per 1000 adults, with men being twice as likely to be affected as women (Fjeld et al., 2019). Risk factors include smoking, strenuous activity, and genetic and environmental factors, with LDH being most common among 30–50-year-olds (Pojskic et al., 2024). Conservative treatment for lumbar disc herniation carries a lower risk of complications than surgery and are favoured by most patients (Deyo et al., 2000). Both surgical and conservative treatments result in similar clinical outcomes over mid-term and long-term follow-up periods (Gugliotta et al., 2016). However, a Swedish study found that surgical treatment is significantly more expensive, with an average cost of $10,311 compared to $2068 for conservative treatment (Hansson and Hansson, 2007).

First line conservative management strategies for LDH include education, lifestyle modification, analgesic medications, physical therapy, and epidural steroid injections (Yang et al., 2020). A key potential advantage of conservative management is the avoidance of the risks and complications associated with surgery (Cook et al., 2021). The WFNS spine committee recommends that in the absence of any red flag symptoms, a combination of activity modification, medication, and physical therapy yields positive outcomes for most patients with lumbar disc herniation (Oertel et al., 2024).

29.66 % of patients undergoing herniated spinal lumbar disc surgery experienced postoperative complications, with the most common being chronic pain and the need for re-surgery (Sehat et al., 2023). This highlights the critical importance of conservative management as a first-line approach for many patients. While conservative therapies have garnered considerable interest, a comprehensive analysis that combines both quantitative and qualitative syntheses is necessary to fully evaluate their effectiveness and compare the relative benefits of specific treatment modalities. Recent meta-analyses addressing conservative management for LDH have often lacked the granularity needed to distinguish between individual strategies or assess their effectiveness across diverse patient subgroups. Our systematic review and meta-analysis aims to bridge this gap, providing a thorough and nuanced evaluation of the existing literature to guide evidence-based decision-making and improve patient care in patients with lumbar disc herniations.

## 2 Methodology

### 2.1 Search strategy and study selection

This systematic review was conducted in accordance with PRISMA guidelines. The search strategy was designed to identify studies on exercise, manual, and traction physiotherapy for the management of lumbar disc herniation (LDH). The search was conducted on May 17, 2024, across five databases, and grey literature: PubMed, MEDLINE, EMBASE, OVID and Scopus. The full search strategy is detailed in Supplementary Material: Table 1. Eligibility criteria followed the PICOS framework (Population, Intervention, Comparison, Outcomes, Study Design). Included studies evaluated conservative treatments for LDH without surgical intervention and reported outcomes such as pain reduction, functional improvement, or quality of life. Exclusion criteria encompassed studies focusing on surgical treatments, thoracic or cervical herniations, paediatric populations, non-English publications, and non-peer-reviewed articles. Further details on inclusion and exclusion criteria are in Supplementary Material: Table 2. The initial screening of titles and abstracts was conducted using Covidence software, allowing for duplicate removal and independent review by five authors (DR, SRN, SGT, HSP, AS). Full-text articles were independently assessed by two reviewers, and any discrepancies were resolved by discussion or consultation with SRN, HSP and SGT.

**Table 1:** Study characteristics of the included studies in this systematic review.

Study	Sample size	Study Type & Design	Country	Level of Evidence	Intervention	Control	Indication for Therapy	Follow-Up Period	Primary and Secondary Outcomes	Definition of Improvement	LTFU	Complications
Taşpınar et al. (2022) (Taşpınar et al., 2023)	52	Randomized Controlled Trial	Turkey	1b	Clinical Pilates Exercises	Normal Daily Routine	Patients diagnosed with LDH, and had lower back and leg pain for 6 weeks.	No follow up	Primary: Pain at rest (cm), General Pain (cm), Pain during exercise (cm) Secondary: Sit and Reach Test, Finger Floor Distance Test, Sit-Bridge Test, Sit-Ups Test, ODI	Statistically significant (p < 0.05) change in any of the outcomes	NR	NR
Danazumi et al. (2021) (Danazumi et al., 2021)	60	Randomized Controlled Trial	Nigeria	1b	SWLM, PINS, SWLM + PINS	No control	Patients diagnosed with unilateral lumbar radiculopathy secondary to lumbar disc herniation	3 months, 6 months, 9 months	VAS leg and back, Roland Morris Disability Questionnaire (RMDQ), Sciatica Bothersomeness Index (SBI).	VAS: A 30 % change is considered clinically significant. RMDQ: A change of 2–3 points (or 8–12 %) is considered clinically significant. SBI: A change of 6.5 points is considered clinically significant	NR	NR
Plaza-Manzano et al. (2020) (Plaza-Manzano et al., 2020)	32	Randomized Controlled Trial	Spain	1b	Neurodynamic Mobilisation + Motor Control Exercises	Motor control Exercises Alone	LBP with confirmed disc herniation and lumbar radiculopathy	Baseline, after 4 and 8 sessions, and 2 months post-intervention.	Pain Intensity (NPRS), RMDQ, SLR test, S-LANSS	Pain (NPRS): ≥2-point reduction. Disability (RMDQ): ≥5-point reduction. Straight Leg Raise (SLR): ≥16° increase. 4o .	NR	NR
Yildirim et al. (2022) (Yildirim and Gultekin, 2022)	48	Prospective Cohort Study	Turkey	2b	Yoga	Patient education only	Females aged 18–50, with imaging confirmed herniation at L4-S1.	Follow-ups at 1 month, 3 months, and 6 months post-treatment.	VAS, ODI, PKE, Schober, LANSS, McTotal, DN4, McLikert	Statistically significant differences between before and after treatment values	5 patients did not adhere to the exercise program	NR
Abdi et al. (2023) (Abdi et al., 2023)	90	Randomized Controlled Trial	Iran	3b	McKinzie Exercises and William's Exercises	Early walking and physiotherapy	Patients with lumbar disc herniation surgery	Baseline, after the 8-week intervention, and 14th postoperative week.	VAS, ODI, TFET, mBST	Statistically different differences between group outcome values	Long term adherence was not properly investigated	Ankle sprain and gluteal sprain
Danazumi et al. (2023) (Danazumi et al., 2023)	40	Randomized Controlled Trial	Nigeria	1b	2 main interventions: Manipulation and Mobilisation	No control	Chronic (>3 months) DHR confirmed through MRI or clinical examinations.	Baseline, 6-, 12-, 26-, and 52-weeks post-randomization.	VAS back, VAS leg, RMDQ, SBI, SFI, TUG, SF-36, GROC	- VAS: ≥2-point decrease - RMDQ: ≥2–3-point decrease - SBI/SFI: ≥6.5-point decrease - TUG: ≥3.4-s improvement - SF-36: ≥10 % improvement GROC: Patient-reported overall improvement	NR	NR
Nikoobakht et al. (2016) (Nikoobakht et al., 2016)	177	Randomized Controlled Trial	Iran	1b	Percutaneous laser disc decompression	Physical therapy, spinal manipulation, NSAIDs, local injections	Lumbar disc herniation	1 year	Primary: VAS, ODI Secondary: SF-36	Reduction in VAS and ODI scores and/or improvement in quality of life.	4 in PDD group, 5 in conservative.	Local anaesthetic-related side effects in PDD group.
Thackeray et al. (2017) (Thackeray et al., 2017)	362	Prospective Cohort Study	USA	2b	Exercise-based physical therapy	Conservative care excluding physiotherapy	Symptomatic lumbar disc herniation	1 year	Primary: SF-36, ODI Secondary: Sciatica Bothersomeness Index, opioid use, self-rated overall improvement	Self-rated improvement or satisfaction on standardised scales.	NR	NR
Kuligowski et al. (2019) (Kuligowski et al., 2019)	37	Prospective Cohort Study	Poland	3b	Pulsed 3D lumbar traction therapy	None	Lumbar disc herniation	Immediate post-therapy	Primary: ODI, NRS Secondary: Passive Lumbar Extension (PLE) and Straight Leg Raise (SLR) tests outcomes	Reduction in ODI and NRS scores and/or improved results in PLE/SLR tests.	NR	NR
Asiri et al. (2020) (Asiri et al., 2020)	25	Prospective Cohort Study	Saudi Arabia	2b	3D lumbar traction therapy	None	Lumbar intervertebral disc prolapse	4 weeks	Primary: VAS, Pain Pressure Threshold (PPT), ODI	Reduction in VAS and ODI scores and/or increase in PPT.	NR	Mild pain and numbness in lower limbs during session (N = 4)
Shokri et al. (2018) (Shokri et al., 2018)	20	Quasi-experimental study	Iran	1b	Lumbar and sacroiliac joint manipulation	None	Chronic lumbar disc herniation with sacroiliac joint hypomobility	1 month	Primary: NRS, ODI Secondary: SIJ mobility tests, SLR and Slump test results.	NRS: ≥20 points ODI: ≥6 points	NR	NR
Ozturk et al. (2006) (Ozturk et al., 2006)	46	Randomized Controlled Trial	Turkey	3b	Continuous lumbar traction with physical therapy	Physical therapy only	Lumbar disc herniation with associated low back pain or sciatica.	Immediately after the 15-session treatment period	Primary: VAS, SLR angle, Herniation size Secondary: Neurological findings	Reduction in herniation index and improvements in VAS, SLR and reflex recovery.	NR	Gastrointestinal side effects from ibuprofen in traction group (N = 2)
Moustafa et al. (2015) (Moustafa and Diab, 2013)	64	Randomized Controlled Trial	Egypt	2b	Lumbar extension traction therapy	Hot packs and interferential therapy only	Chronic unilateral lumbosacral radiculopathy with lumbar lordotic angle <39°	6 months	Primary: Absolute rotatory angle (lumbar lordosis), NPRS for back and leg pain, ODI Secondary: Lumbar flexibility (Modified Schober test), H-reflex latency and amplitude.	NR	Traction: 2 Control: 4	NR
Unlu et al. (2008) (Unlu et al., 2008)	60	Randomized Controlled Trial	Turkey	2b	Either: 1) Intermittent motorized lumbar traction. 2) Low-power laser 3) Continuous ultrasound	Each intervention served as a comparator	Lumbar disc herniation with associated low back and radicular leg pain.	3 months	Primary: VAS for low back and leg pain, Roland Disability Questionnaire (RDQ) and Modified Oswestry Disability Questionnaire (MODQ), Herniation size	Reduction in pain (VAS), disability (RDQ, MODQ), and herniation size on MRI.	NR	NR
Hahne et al. (2017) (Hahne et al., 2017)	54	Randomized Controlled Trial	Australia	1b	Individual Functional Restoration	Advice	Lumbar disc herniation with associated radiculopathy	52 weeks	Primary: ODI for activity limitation, NRS for back and leg pain. Secondary: Patient satisfaction, Sciatica frequency and bothersomeness, Health-related quality of life	ODI: ≥10-point reduction NRS (pain): ≥2-point reduction Global rating of change: “much improved” or better Satisfaction: “very satisfied”	Advice: 1	NR
Weinstein et al. (2006) (Weinstein et al., 2006)	743	Prospective Cohort Study	USA	2b	Open discectomy	Non-operative care e.g. physical therapy, epidural injections, NSAIDs	Persistent sciatica from lumbar disc herniation.	2 years	Primary: SF-36 Bodily Pain, SF-36 Physical Function, ODI Secondary: Sciatica Bothersomeness Index, Satisfaction, Self-rated Improvement	SF-36 scales: ≥10-point improvement ODI: ≥8–12-point reduction	Surgery: 18 Non-op: 6	Surgery: Dural tears (2 %), Reoperation rate (9 %).
Svensson et al. (2014) (Svensson et al., 2014)	41	Prospective Cohort Study	Sweden	2b	Structured physiotherapy including McKenzie Method and trunk stabilization exercises	None	Lumbar disc herniation with leg pain or neurological disturbance	2 years	Primary: VAS for leg pain, ODI for disability. Secondary: VAS for back pain, Tampa Scale for Kinesiophobia (TSK), Health-related quality of life, Zung Self-Rating Depression Scale (ZDS)	ODI: Score of <20 % defined as minimal or no disability. For leg pain: VAS <10 mm defined as no pain.	5 (4 underwent surgery, 1 missed follow-up)	NR
Isner-Horobeti et al. (2016) (Isner-Horobeti et al., 2016)	17	Randomized Controlled Trial	France	2b	LT50: High-force lumbar traction LT10: Low-force lumbar traction	None	Acute lumbar sciatica	28 days	Primary: VAS for radicular pain Secondary: EIFEL score for functional impairment, Schober-Macrae test for lumbar spine mobility, patient satisfaction, SLR test, Finger-to-Toe test	Significant VAS score reduction and/or increased SLR test angle.	NR	NR
Ehrler et al. (2016) (Ehrler et al., 2016)	68	Prospective Cohort Study	Switzerland	2b	Spinal manipulative therapy	None	Acute or chronic low back pain with moderate-to-severe leg pain	12 months	Primary: Patients' Global Impression of Change (PGIC) Secondary: NRS for back and leg pain, Oswestry Pain and Disability Questionnaire (OPDQ) for disability.	Clinically relevant improvement defined as a response of "much better" or "better" on the PGIC scale.	NR	NR
Kumari et al. (2021) (Kumari et al., 2021)	45	Randomized Controlled Trial	India	1b	Group A: One-fifth bodyweight traction Group B: One-third bodyweight traction Group C: One-half bodyweight traction.	Comparison made between different traction force groups	Lumbar prolapsed intervertebral disc, positive unilateral SLR test and one additional neurological sign	Immediate post-intervention assessment	Primary: SLR Range of Motion ROM), Pain intensity with VAS	Significant VAS score reduction and/or increased SLR test angle.	NR	NR
Leemann et al. (2014) (Leemann et al., 2014)	148	Prospective Cohort Study	Switzerland	2b	Spinal manipulative therapy	None	Lumbar disc herniation with radiculopathy	1 year	Primary: Patients' Global Impression of Change (PGIC) Secondary: NRS for back and leg pain, ODI for disability.	Clinically relevant improvement defined as a response of "much better" or "better" on the PGIC scale.	23	NR
Choi et al. (2022) (Choi et al., 2022)	60	Randomized Controlled Trial	South Korea	1b	Nonsurgical Spinal Decompression Therapy	Pseudodecompression therapy	Lumbar disc herniation, pain duration of 4 weeks–3 months and VAS score ≥4	3 months	Primary: VAS for lower back and leg pain, Korean-ODI for disability Secondary: Change in Herniation Index (HI)	Significant reduction in VAS scores for pain and K-ODI scores for disability.	Decompression: 4 Pseudodecompression: 13	NR
He et al. (2006) (He et al., 2006)	60	Randomized Controlled Trial	China	2b	Herbal magnetic corset	Traction, electrotherapy and massage.	Lumbar disc herniation with lower back and radicular leg pain, and neurological signs	4 weeks	Primary: VAS for pain reduction, Lumbar function improvement (Lumbar Disease Grade) Secondary: Improvement index	Significant reduction in VAS score and improvement in lumbar function	Corset: 1 Traction: 1	NR
Annen et al. (2016) (Annen et al., 2016)	72	Prospective Outcomes Study	Switzerland	2b	Spinal manipulative therapy	None	Symptomatic lumbar disc herniation	1 year	PGIC, NRS, ODI	PGIC of “better” or “much better”	NR	NR
Gugliotta et al. (2016) (Gugliotta et al., 2016)	370	Prospective Cohort Study	Switzerland	2b	Open discectomy	Conservative treatment: physical therapy, pharmacological treatment, home-based exercises	Lumbar disc herniation causing sciatica	2 years	SF-36 and NASS	≥50 % reduction in NASS back scores	NR	NR
Salfinger et al. (2015) (Salfinger et al., 2015)	94	Randomized Controlled Trial	Austria	2b	tNMR	Sham treatment	Lumbar radicular syndrome caused by lumbar disc herniation	3 months	VAS, SF-36, RMDQ	No specific threshold other than reduction in scores	14 patients (surgical needs or personal reasons)	NR
Peul et al. (2008) (Peul et al., 2008)	283	Randomized Controlled Trial	Netherlands	1b	Early lumbar discectomy	6 months of prolonged conservative care	Sciatica persisting for 6–12 weeks caused by lumbar disc herniation	2 years	RDQ, VAS, Global Perceived Recovery	“Satisfactory Recovery” defined as complete or almost complete resolution of symptoms	23 participants (crossed over)	Surgical complications in 1.6 %: 2 dural tears, 1 wound haematoma
Ghaderi Niri et al. (2024) (Ghaderi Niri et al., 2024)	92	Observational Cohort Study	Iran	2b	Physiotherapy program	General exercise	None	4 weeks	ODI, RMDQ, QBPDS	ODI: ≥13 points. RMDQ: ≥5.5 points. QBPDS: ≥14.5 points.	NR	NR
Khanzadeh et al. (2020) (Khanzadeh et al., 2020)	30	Quasi-Experimental Study	Iran	2b	Lumbar traction therapy	None	Lumbar disc herniation (L4-L5 and L5-S1).	8 weeks	VAS, intervertebral disc height, Herniation Index	General reduction in scores	5 participants (3 from suspension 2 from control)	NR
Tarcău et al. (2022) (Tarcău et al., 2022)	60	Prospective Cohort Study	Romania	1b	Electrotherapy, Hydrotherapy and Individualized Physical Therapy	Electrotherapy only treatment	Chronic lumbar disc protrusion with symptoms >3 months	6 months	VAS. SF-MPQ measuring pain and ODI measuring disability	≥10 % reduction in SF-MPQ and >30 % improvement in ODI	None	NR
Ye et al. (2015) (Ye et al., 2015)	63	Controlled Clinical Trial	China	1b	Lumbar Spine stabilization exercises	Standard physiotherapy treatments	Lumbar disc herniation	6 months	NPRS, ODI, SF-12	>1 point reduction in NPRS and >9 point reduction in ODI	6 participants	NR
Bello et al. (2019) (Bello et al., 2019)	40	Randomized Control Trial	Nigeria and South Africa	2b	Dowling Manual Therapy technique	Mulligan Manual Therapy technique	18-65, unilateral radiculopathy, pain in the distribution of the sciatic nerve	Baseline, Week 4, Week 8	Primary: Visual Analog Scale, Roland-Morris Disability Questionnaire Secondary: SF-36, sciatica bothersomeness (Sciatica Bothersomeness Index), sciatica frequency (Sciatica Frequency Index), and general perception of recovery (Global Rating of Change Scale)	Statistically significant (p < 0.05) change in any of the outcomes. No specific definition of improvement.	6 participants (lost to follow-up)	NR
Choi et al. (2015) (Choi et al., 2015)	30	Randomized Control Trial	Japan	2b	Spinal Decompression	General Traction	Chronic Lumbar Pain due to disc herniation	Baseline, Week 4	Visual Analog Scale (VAS) Oswestry Disability Index (ODI), Straight Leg Raise (SLR)	Statistically significant (p < 0.05) change in any of the outcomes.	NR	NR
Iosub et al. (2023) (Iosub et al., 2023)	77	Randomized Control Trial	Taiwan	1b	Vojta therapy and Conservative Physical Therapy	Physical Therapy	30–75 years, an MRI-confirmed diagnosis of lumbar disc herniation, presence of low back and/or leg pain due to disc herniation	Baseline, Week 2	Primary: Visual Analog Scale (VAS) Oswestry Disability Index (ODI) Secondary: hip flexion ability, trunk lateral flexion, qualitfy of life measured with FTF, TRLF, TLLF, HF, MSTFF, MSTRLF, MSTLLF and NHP for HRQL (health-related quality of life).	Statistically significant (p < 0.05) change in any of the outcomes (VAS and ODI)	NR	NR
Singh et al. (2022) (Singh and Malik, 2022)	88	Randomized Control Trial	Poland	2b	Group 1: Spinal Mobilization with Leg Movement (SMWLM) Group 2: High-Velocity Low Amplitude (HVLA) thrust Group 3: Neural Mobilization (NM)	Control Treatment (CT) group	18-50 with lumbar pain, limited range of motion, unilateral radiating pain	Baseline, Week 6	Visual Analog Scale (VAS) Oswestry Disability Index (ODI), Straight Leg Raise (SLR)	Visual Analog Scale (VAS): MCID = ≥2-point reduction. Oswestry Disability Index (ODI): MCID = ≥10-point reduction. Straight Leg Raise Range of Motion (SLR ROM): Minimal Detectable Change (MDC) = ≥5.7-degree increase.	NR	NR
Lee et al. (2020) (Lee et al., 2020)	40	Randomized Control Trial	Lithuania	2b	Lumbar lordotic curve-controlled traction	Traditional Traction	Disc herniation in lumbar spine with lower back pain and/or sciatic symptoms lasting more than 3 months	Baseline, Week 5, Week 6	Visual Analog Scale (VAS) Oswestry Disability Index (ODI) Roland-Morris Disability Questionnaire (RMDQ) Morphological changes in lumbar spine (MRI)	Statistically significant (p < 0.05) change in any of the outcomes, improvement not defined.	NR	NR
França et al. (2019) (França et al., 2019)	40	Randomized Control Trial	US	2b	Motor Control Training	TENS – standard electrotherapy	18–60 yrs with LDH diagnosis, associated with both low back and leg pain and diagnosed through MRI or computed tomography	Baseline, Week 8	Visual Analog Scale (VAS), Oswestry Disability Index (ODI), Transversus Abdominis (TrA) activation	Statistically significant (p < 0.05) change in any of the outcomes	NR	NR
Singh et al. (2019) (Singh, 2019)	30	Randomized Control Trial	Turkey	2b	Supine Lumbar Traction with infrared	Prone Lumbar Traction with infrared	Patient diagnosed with prolapsed disc	Baseline, Week 1, Week 6	Visual Analogue Scale, Modified Oswestry Questions	VAS decrement has meaningful difference (P-value<0.001). ODQ decrement has meaningful difference (p < 0.05).	NR	NR
Murat et al. (2018) (Murat et al., 2018)	61	Randomized Control Trial	Turkey	2b	Bodyweight Traction	Low-load Traction	Lumbar disc herniation with 2 week–3 months of pain, confirmed by MRI	Baseline, Week 2, Week 6	Visual Analog Scale, Oswestry Disability Index, Roland Morris Disability Questionnaire, SF-36	Statistically significant (p < 0.05) change in any of the outcomes	Total 8 lost to follow up: 4 patients excluded due to adverse effects. 2 patients excluded due to non-adherence. 2 patients loss to follow up	NR
Koçak et al. (2017) (Koçak et al., 2017)	48	Randomized Control Trial	Turkey	2b	Conventional Motorized Traction	Non-surgical Spinal Decompression	Disc herniation confimed by MRI	Baseline, Week 6	Visual Analog Scale, Beck Depression Inventory, Oswestry Disability Index, SF-36	Statistically significant (p < 0.001) change in any of the outcomes For some of	NR	NR
Gülşen et al. (2018) (Gŭlşen et al., 2018)	210	Randomized Control Trial	Italy	2b	Group 1: Hot Pack Group 2: TENS Group 3: Ultrasound	No control	20-65 diagnosed with clinical examination and radiological findings of LDH from at least 6 months	Baseline, Week 4	Oswestry Disability Index, Roland Morris Disability Questionnaire, Visual Analog Scale	Statistically significant (p < 0.05) change in any of the outcomes	NR	NR
Keles et al. (2017) (Keles et al., 2017)	52	Randomised Control Trial	Netherlands	2b	Kinesio Taping	Placebo	18-45, LDH confirmed by history, examination and MRI, lower back pain for at least 3 months	Baseline, Week 1, Week 2, Week 3, Week 6, Week 12	Numeric Rating Scale, Health Assessment Questionnaire, Oswestry Disability Index	Statistically significant (p < 0.05) change in any of the outcomes and 95 % confidence level	NR	NR
Luijsterburg et al. (2007) (Luijsterburg et al., 2007)	135	Randomized Controlled Trial	Netherlands	1b	Physical therapy added to GP care	GP care alone (education/advice, medication as needed)	Sciatica <6 weeks, radiating leg pain with neurological signs	Baseline, 1 year (3, 6, 12, and 52 weeks)	Global Perceived Effect (GPE), EQ-5D quality of life, direct and indirect costs, ICER	“Completely recovered” or “much improved” on 7-point GPE scale	13 % at 1 year (117/135 completed)	NR

In Table 1, the study characteristics of all included studies are presented, detailing the sample size, study type and design, country of origin, interventions and controls, indications for therapy, follow-up periods, primary and secondary outcomes, definitions of improvement, loss to follow-up rates, and reported complications. **RCT**: Randomized Controlled Trial; **VAS**: Visual Analog Scale; **ODI**: Oswestry Disability Index; **SLR**: Straight Leg Raise; **CI**: Confidence Interval; **LTFU**: Loss to Follow-Up; **NR**: None reported.

**Table 2:** Detailed summary of the results from each of the included studies in this systematic review (including studies that focused only on exercise).

Study	Treatment	Methodology	Sample Size: I	Sample Size: C	VAS Difference in %	ODI Difference in %	SF-36 Difference in %	Adverse Effects	Key Findings	Risk of Bias
Taşpınar et al. (2022)	Clinical Pilates Exercise (CPE)	Randomized controlled trial with 54 participants. Compared clinical Pilates exercises to no treatment/normal daily routine.	27	27	54.9	48.1	N/A	NR	Clinical Pilates: Reduced pain (rest, general, exercise), improved functionality (ODI), spinal flexibility, trunk endurance, and quality of life (SF-36).	ROB-2
										D1: Low
										D2: Low
										D3: Low
										D4: Low
										D5: Low
										D6: Low
Asiri et al. (2020)	Custom lumbar traction therapy designed with a patient-specific 3D model to optimize treatment efficacy.	Twenty-five patients with lumbar disc prolapse underwent 12 sessions of customized three-dimensional lumbar traction over 4 weeks, with pain and disability measured before, during, and after treatment.	25	N/A	N/A	50.0	N/A	NR	The study showed that 12 sessions of Patient-Specific 3D Lumbar Traction significantly reduced pain (VAS: 8.56 to 3.22) and disability (ODI: 53.55 %–31.36 %) and improved pressure pain threshold (PPT: 0.70–1.64 kg/cm^2^) over 4 weeks, with no major adverse effects. Custom machine adjustments tailored to patients contributed to these results.	ROBINS-I:
										D1: Moderate
										D2: Low
										D3: Low
										D4: Low
										D5: Low
										D6: Low
										D7: Low
										D8: Low
Yildirim et al. (2022)	The yoga program included poses such as cat-cow, bridge, triangle, plank, and warrior. Progression was based on complexity, range of motion, and intensity.	Prospective Cohort Study comparing SMWLM, PINS, and their combination.	24	24	38.3	N/A	N/A	Ankle and gluteal sprain (N = 2)	Yoga: Decreased pain (VAS, ODI), improved flexibility, core strength, and functional outcomes, with high adherence and safety.	ROBINS-I
										D1: Moderate
										D2: Moderate
										D3: Moderate
										D4: Low
										D5: Low
										D6: Moderate
										D7: Moderate
										D8: Moderate
Abdi et al. (2023)	Both groups performed home-based exercises with instructions provided via face-to-face training, videos, and posters. Follow-up every two weeks.	Single blind randomized control trial with 90 participants. Testing McKinzie Exercises and William's Exercises compared to early walking and physiotherapy as a control	30	30	N/A	20.3	N/A	NR	McKenzie vs. Williams Exercises: Both reduced pain/disability (ODI), with McKenzie showing greater improvements; enhanced trunk endurance differently.	ROB-2
										D1: Low
										D2: Low
										D3: Low
										D4: Low
										D5: Low
										D6: Low
Thackeray et al. (2017)	Exercise-based physical therapy compared to usual care.	Cohort Study: Analyzed lumbar disc herniation outcomes with/without physical therapy in 13 spine centers using SF-36 and ODI measures.	143	219	54.4	N/A	N/A	NR	Physical Therapy Study: No significant difference in outcomes between therapy and no therapy; highlighted nonsurgical treatment diversity and need for standardization.	ROBINS-I:
										D1: Moderate
										D2: Moderate
										D3: Moderate
										D4: Moderate
										D5: Moderate
										D6: Low
										D7: Moderate
										D8: Moderate
Svensson et al. (2014)	Comparing surgical treatment to structured physiotherapy	Cohort Study: Evaluated a 9-week physiotherapy program (MDT + trunk stabilization) in 41 MRI-confirmed lumbar disc herniation patients with follow-ups at 3, 12, and 24 months.	41	N/A	1.19	2.18	N/A	NR	Physiotherapy Program: Reduced disability/pain (ODI, VAS), minimized surgery rates (7 % in 12 months), and improved psychosocial factors like Kinesiophobia and self-efficacy.	ROBINS-I:
										D1: Moderate
										D2: Low
										D3: Moderate
										D4: Moderate
										D5: Low
										D6: Moderate
										D7: Low
										D8: Moderate
Luijsterburg et al. (2007)	Physical therapy (exercise therapy, education/advice) added to GP care	Randomized controlled trial with economic evaluation in 135 patients with acute lumbosacral radicular syndrome (sciatica). Compared PT + GP care vs GP care alone.	67	68	N/A	N/A	N/A	NR	PT + GP care led to higher perceived recovery (79 % vs 56 % at 1 year), but no improvement in quality of life (EQ-5D). Costs were higher in the intervention group, and PT was not cost-effective compared to GP care alone.	ROB-2
										D1: Low
										D2: Some concerns
										D3: Low
										D4: Low
										D5: Low
										D6: Low

In Table 2, the findings of studies focusing on exercise-based therapies for lumbar disc herniation are summarized. The variables extracted include treatment methodologies, sample sizes, outcome measures (VAS, ODI, SF-36), adverse effects, and main conclusions. **I**: Intervention; **C:** Control**; N/A:** Not applicable; **NR:** Not reported; **RoB-1**: Risk of Bias 1 Tool **RCT**: Randomized Controlled Trial; **VAS**: Visual Analog Scale; **ODI**: Oswestry Disability Index; **SF-36**: Short Form Health Survey; **QoL**: Quality of Life; **CI**: Confidence Interval; **ROBINS-I**: Risk of Bias in Non-Randomised Studies of Interventions; **RoB-1**: Risk of Bias 1 Tool.

### 2.2 Objectives

This review sought to answer three key research questions:

1. What are the relative efficacy and effect sizes of the most common conservative physiotherapy modalities, exercise therapy, manipulation therapy, and traction therapy, for managing lumbar disc herniation?
2. What factors contribute to variability in treatment effects?
3. Are there specific patient subgroups that may benefit more from one physiotherapy modality over another?

### 2.3 Data extraction and quality assessment

Data extraction was performed manually using a standardized Excel spreadsheet to capture study characteristics, interventions, patient demographics, and outcomes. The extracted data were organized into tables, with results summarized in the main manuscript and supplementary materials (Table 1, Table 2, Table 3, Table 4, Supplementary Material: Table 3). The quality of included studies was assessed using the Risk of Bias in Non-Randomised Studies – of Interventions (ROBINS-I) tool, which evaluates potential biases across seven domains (Sterne et al., 2016). For randomised studies, the Risk of Bias-2 tool was used (RoB-2) (Sterne et al., 2019). Discrepancies in bias assessments were resolved through consensus by a third reviewer. In addition, the Oxford Centre of Evidence-Based Medicine (OCEBM) Levels of Evidence framework was applied to classify the methodological quality and evidence levels of the included studies (Durieux et al., 2013). Furthermore, the GRADE (Grading of Recommendations, Assessment, Development, and Evaluations) framework was employed to assess the overall quality of evidence, emphasizing the balance of benefits and harms for clinical decision-making (Guyatt et al., 2008). The full risk of bias scoring for ROBINS-I, RoB-2, OCEBM and GRADE can be viewed in Supplementary Material: Tables 4–7, respectively.

**Table 3:** Detailed summary of the results from each of the included studies in this systematic review (including studies that focused only on manipulation).

Study	Treatment	Methodology	Sample Size: I	Sample Size: C	VAS Difference in %	ODI Difference in %	SLR Difference in %	Adverse Effects	Key Findings	Risk of Bias
Plaza-Manzano et al. (2020)	Motor Control Exercises (MCE): Core strengthening exercises, with or without added nerve mobilization (NDM) targeting the sciatic nerve.	RCT: 32 patients with lumbar radiculopathy were assessed for pain, disability, nerve symptoms, flexibility, and sensitivity at baseline, mid-treatment, 8 weeks, and 2 months post-treatment. r.	16	16	78.77	N/A	N/A	NR	Both groups improved in pain and disability, but adding NDM didn't make a big difference. NDM led to slightly better improvement in nerve symptoms and flexibility. Overall, the added benefit of NDM was small and not clinically significant	ROB-2
										D1: Moderate
										D2: Low
										D3: Low
										D4: Low
										D5: Moderate
										D6: Moderate
Shokri et al. (2018)	Spinal Manipulative Therapy (SMT): Includes lumbar rotation manipulation and sacroiliac joint thrusts to improve mobility and reduce pain.	Quasi-Experimental Study: 20 patients with MRI-confirmed LDH and SIJ hypomobility were evaluated for pain and disability at baseline, after five sessions, and one month post-treatment.	20	N/A	N/A	100.00	N/A	NR	SMT reduced back/leg pain and disability, but changes were not clinically significant. SIJ hypomobility tests improved in 95 % of patients, but SLR and slump test results did not.	ROBINS-I
										D1: Moderate
										D2: Moderate
										D3: Low
										D4: Low
										D5: Low
										D6: Moderate
										D7: Low
										D8: Moderate
Hahne et al. (2017)	Functional restoration program (10 sessions) + advice vs. advice alone (2 sessions).	RCT with 54 participants, lumbar disc herniation with radiculopathy. Outcomes: ODI, back and leg pain (NRS), assessed at 5, 10, 26, and 52 weeks.	28	26	N/A	44.29	N/A	NR	Functional restoration reduced activity limitation at 10 and 52 weeks. Back pain improved at 10 weeks; no sustained leg pain differences.	ROB-2
										D1: Low
										D2: Low
										D3: Low
										D4: Low
										D5: Low
										D6: Low
Ehrler et al. (2016)	High-velocity, low-amplitude spinal manipulation (SMT) tailored to the herniation's MRI-confirmed location (paramedian or foraminal) and applied at the specific herniation level.	A prospective cohort study with 68 patients receiving SMT for MRI-confirmed lumbar disc herniations. Outcomes (pain, disability, and patient impression of change) were assessed at baseline, 2 weeks, 1 month, 3 months, 6 months, and 1 year, with results analyzed by herniation type and location.	68	N/A	N/A	N/A	N/A	NR	90.5 % achieved significant improvement by 3 months, sustained at 1 year (88 %). Acute cases improved faster; chronic cases showed steady progress. Significant reductions in pain and disability (p < 0.0001). No adverse events reported; 3 patients required surgery.	ROBINS-I
										D1: Moderate
										D2: Moderate
										D3: Low
										D4: Low
										D5: Moderate
										D6: Low
										D7: Low
										D8: Low
Leemann et al. (2014)	. High-velocity, low-amplitude spinal manipulation therapy (SMT) tailored to herniation type (intraforaminal or paramedian) to reduce nerve pressure, restore mobility, and alleviate pain.	Prospective cohort study of 148 patients (18–65 years) with MRI-confirmed lumbar disc herniations, divided into acute (<4 weeks) and chronic (>12 weeks) groups. Outcomes (pain, disability, and improvement) were assessed at baseline, 2 weeks, 1, 3, and 6 months, and 1 year.	148	N/A	74.30	N/A	N/A	NR	90.5 % showed significant improvement by 3 months, sustained at 1 year (88 %). Acute cases improved faster (94.5 % by 3 months); chronic cases showed steady progress (89.2 % at 1 year). Pain and disability significantly reduced across all time points (p < 0.0001). No adverse events reported; 3 patients opted for surgery.	ROBINS-I
										D1: Low
										D2: Low
										D3: Low
										D4: Low
										D5: Low
										D6: Low
										D7: Low
										D8: Low
Annen et al. (2016)	High-velocity, low-amplitude spinal manipulative therapy (SMT) targeting lumbar disc herniations based on MRI findings, including Modic changes (MCs).	Prospective outcomes study with 72 patients (18–65 years) with symptomatic, MRI-confirmed lumbar disc herniations. Outcomes (pain, disability, and improvement) were assessed at multiple intervals up to 1 year, comparing results based on the presence and type of Modic changes.	72	N/A	31.62	N/A	N/A	NR	MC-positive patients showed faster initial improvement in leg pain and disability. At 1 year, MC-negative patients had better outcomes than those with Modic type I changes, who were prone to relapses. Modic type II patients had the most favorable long-term results.	ROBINS-I
										D1: Low
										D2: Low
										D3: Moderate
										D4: Moderate
										D5: Low
										D6: Low
										D7: Moderate
										D8: Moderate
Singh et al. (2022)	Three manual therapies (SMWLM, HVLA, NM) combined with stabilization exercises, traction, and interferential therapy.	Double-blind RCT with 88 patients divided into four groups (SMWLM, HVLA, NM, Control). Outcomes measured included pain, disability, and neural mobility.	66	22	74.69	58.51	92.42	NR	SMWLM showed the greatest improvements in pain (VAS: 6.05), disability (ODI: 15.65), and neural mobility (SLR: 15.06). HVLA and NM also improved outcomes but were less effective. Control group saw minimal changes, confirming manual therapy efficacy. Benefits were sustained at follow-up.	ROB-2
										D1: Low
										D2: Moderate
										D3: Low
										D4: Moderate
										D5: Low
										D6: Moderate
Danazumi et al. (2023)	Two interventions for chronic lumbar disc herniation with radiculopathy: SMT: High-velocity spinal manipulation. MOB: Mulligan's mobilization with leg movement. Both groups received Neurodynamic Mobilization (NM).	Single-blind RCT with 40 participants split into SMT and MOB groups. Outcomes (pain, activity limitation, mobility, quality of life) were assessed at baseline and multiple follow-ups (6 weeks–1 year).	20	20	N/A	N/A	N/A	NR	The MOB + NM group showed superior improvements in pain, mobility, activity, and quality of life across all time points, with significant short-term (6 weeks) and sustained long-term (52 weeks) benefits. SMT group had 40 % lower improvement odds at 12 weeks. 4o	ROB-2
										D1: Low
										D2: Low
										D3: Low
										D4: Low
										D5: Low
										D6: Low

In Table 3, the findings of manipulation-based therapies for lumbar disc herniation are summarized. Variables include treatment methods, sample sizes, outcome measures (VAS, ODI, SLR), adverse effects, and primary conclusions. **RCT**: Randomized Controlled Trial; **VAS**: Visual Analog Scale; **ODI**: Oswestry Disability Index; **QoL**: Quality of Life; **SLR**: Straight Leg Raise; **CI**: Confidence Interval; **MRI**: Magnetic Resonance Imaging.**ROBINS-I**: Risk of Bias in Non-Randomised Studies of Interventions; **RoB-1**: Risk of Bias 1 Tool.

**Table 4:** Detailed summary of the results from each of the included studies in this systematic review (including studies that focused only on traction).

Study	Treatment	Methodology	Sample Size: I	Sample Size: C	VAS difference in %	ODI Difference in %	SLR Difference in %	Adverse Effects	Key Findings	Risk of bias
Asiri et al. (2020)	Custom lumbar traction therapy designed with a patient-specific 3D model to optimize treatment efficacy.	Twenty-five patients with lumbar disc prolapse underwent 12 sessions of customized three-dimensional lumbar traction over 4 weeks, with pain and disability measured before, during, and after treatment.	25	N/A	N/A	50.0	N/A	NR	The study found that 12 sessions of Patient-Specific Three-Dimensional Lumbar Traction (PS3DLT) significantly reduced pain (VAS decreased from 8.56 to 3.22) and improved functional disability (ODI reduced from 53.55 % to 31.36 %) in patients with lumbar disc prolapse over 4 weeks. Pressure Pain Threshold (PPT) also increased notably from 0.70 kg/cm^2^ to 1.64 kg/cm^2^, reflecting reduced pain sensitivity. The customized adjustments of the traction machine, tailored to each patient's specific needs, contributed to these improvements, with no major adverse effects reported.	ROBINS-I:
										D1: Moderate
										D2: Low
										D3: Low
										D4: Low
										D5: Low
										D6: Low
										D7: Low
										D8: Low
Ozturk et al. (2005)	Continuous lumbar traction combined with physical therapy (hot packs, ultrasound, and diadynamic currents) and medications (ibuprofen and muscle relaxants).	A randomized controlled trial with 46 lumbar disc herniation patients divided into a traction group (with therapy) and a control group (therapy only). Outcomes assessed pain, mobility, neurological deficits, and herniation size via CT scans.	24	22	61.90	N/A	61.83	Mild discomfort	Continuous lumbar traction is effective in reducing pain and improving straight leg raise in lumbar disc herniation patients.	ROB-2
										D1: Moderate
										D2: Low
										D3: Low
										D4: Low
										D5: Low
										D6: Moderate
Moustafa et al. (2012)	Lumbar extension traction plus conventional therapy (hot packs, interferential therapy) was compared to conventional therapy alone for 10 weeks.	Randomized controlled trial with 64 patients with L5–S1 disc herniation, assessed at baseline, 10 weeks, and six months for pain, disability, lumbar alignment, and neurological function. l	32	32	62.90	38.89	N/A	NR	Traction significantly improved spinal alignment, reduced pain and disability, and enhanced mobility and neurological function compared to conventional therapy, with sustained benefits at six months.	ROB-2
										D1: Low
										D2: Low
										D3: Low
										D4: Low
										D5: Low
										D6: Low
Choi et al. (2022)	Nonsurgical Spinal Decompression Therapy (NSDT) delivered over 8 weeks aimed to reduce herniation via negative disc pressure, compared to pseudodecompression (control).	Randomized controlled trial with 60 subacute lumbar herniation patients measuring pain (VAS), disability (K-ODI), and herniation size (MRI).	30	30	83.40	N/A	N/A	NR	NSDT reduced herniation size significantly (−27.6 % vs. −7.1 %, p = 0.017). Greater improvements in leg pain and function with NSDT (p < 0.05). 26.9 % of NSDT patients had >50 % herniation reduction vs. none in controls (p = 0.031).	ROB-2
										D1: Low
										D2: Low
										D3: Low
										D4: Low
										D5: Low
										D6: Low
He et al. (2006)	Standard care (traction, electrotherapy, massage) with or without a herbal magnetic corset.	RCT with 60 patients; outcomes (pain and function) measured at baseline, 1, 2, and 4 weeks l	30	30	30.65	32.56	16.54	NR	Both groups improved in pain and function, but the experimental group showed significantly greater improvements, especially at 2 and 4 weeks. The herbal magnetic corset enhanced recovery compared to standard care alone.	ROB-2
										D1: Low
										D2: Low
										D3: Low
										D4: Low
										D5: Low
										D6: Low
Choi et al. (2015)	Spinal Decompression Therapy (SDTG): Gradual traction to reduce nerve root pressure. General Traction Therapy (GTTG):	Design: Comparative, non-randomized trial with 30 patients split into SDTG and GTTG groups. Measures: Pain (VAS), disability (ODI), and straight leg raise (SLR), assessed pre- and post-treatment.	15	15	47.89	36.84	N/A	NR	Both therapies significantly improved pain, disability, and SLR. No significant difference between SDTG and GTTG outcomes. Both are effective and can be chosen based on patient preferences and resources.	ROB-2
										D1: Moderate
										D2: Moderate
										D3: Low
										D4: Low
										D5: Low
										D6: Moderate
Iosub et al. (2023)	Group A: Vojta therapy plus standard physical therapy. Group B: Standard physical therapy alone.	Randomized control trial study with 77 lumbar disc herniation patients, randomized into two groups. Pain and disability were primary outcome	38	39	76.56	58.54	N/A	NR	Vojta therapy significantly reduced pain and disability compared to standard therapy alone.	ROB-2
										D1: Moderate
										D2: Low
										D3: Low
										D4: Low
										D5: Low
										D6: Moderate
França et al. (2019)	Compared Motor Control Training (MCT) (core stabilization exercises) with TENS (pain management) over 8 weeks (16 sessions).	Single-blind RCT with 40 MRI-confirmed lumbar disc herniation patients split into MCT or TENS groups; outcomes assessed for pain, disability, and core muscle activation.	20	20	48.20	35.55	N/A	NR	MCT significantly outperformed TENS in reducing pain, improving disability, and enhancing core muscle activation.	ROB-2
										D1: Low
										D2: Low
										D3: Low
										D4: Low
										D5: Low
										D6: Low
Gǔlşen et al. (2018)	Three groups: physiotherapy, physiotherapy + traction, and home exercise.	RCT with 210 lumbar disc herniation patients over 4 weeks; outcomes measured pain and disability.	75	70	N/A	0.00	N/A	NR	All groups improved; traction added no extra benefit; home exercise was effective alone.	ROB-2
										D1: Low
										D2: Moderate
										D3: Low
										D4: Low
										D5: Low
										D6: Moderate
Keles et al. (2017)	Kinesio Taping (KT) versus placebo taping alongside home exercises for lumbar disc herniation.	Randomized, double-blind trial with 60 participants, evaluating pain, disability, and analgesic use over 12 weeks.	29	23	N/A	19.64	71.00	Skin irritation	KT showed slight improvements in pain, disability, and reduced analgesic use compared to placebo, but differences were modest.	ROB-2
										D1: Low
										D2: Low
										D3: Low
										D4: Low
										D5: Low
										D6: Low

In Table 4, the results of studies examining traction therapies for lumbar disc herniation are presented. The table includes details on sample sizes, traction methods, outcome measures (VAS, ODI, SLR), adverse effects, and main findings. **RCT**: Randomized Controlled Trial; **VAS**: Visual Analog Scale; **ODI**: Oswestry Disability Index; **SLR**: Straight Leg Raise; **MRI**: Magnetic Resonance Imaging; **SD**: Standard Deviation; **CI**: Confidence Interval; **ROBINS-I**: Risk of Bias in Non-Randomised Studies of Interventions; **RoB-1**: Risk of Bias 1 Tool.

### 2.4 Outcomes

The primary outcomes assessed in this review were pain reduction and functional improvement, as reported in the included studies. Pain was most commonly measured using validated scales such as the Visual Analog Scale (VAS) and Numeric Rating Scale (NRS), while disability and functional status were typically evaluated using the Oswestry Disability Index (ODI) and the Roland–Morris Disability Questionnaire (RMDQ). For the quantitative synthesis, these measures were standardized using the Standardized Mean Change (SMC), which reflects the magnitude of within-group change from baseline to follow-up. A positive SMC indicates an improvement in outcomes from baseline.

### 2.5 Statistical analysis

Data preparation was performed using SPSS (IBM, USA) Version 28.0.0.0 (Corp, 2021). Subsequently, R software (version 4.4.3) was used for statistical analysis and forest plot synthesis, utilizing the meta and metafor packages (Team RC). A random-effects model meta-analysis was conducted to calculate the pooled SMC for the three primary conservative therapy modalities: exercise therapy, manipulation therapy, and traction therapy. Forest plots were generated to visualize individual study effect sizes, their confidence intervals (95 % CI), and the overall pooled estimates. Heterogeneity was assessed using the I^2^ statistic and corresponding p-values, with I^2^ > 50 % indicating substantial heterogeneity. Publication bias was evaluated using funnel plots and Egger's test, with statistical significance set at p < 0.05. An asymmetry in the funnel plot and significant Egger's test results were interpreted as evidence of potential publication bias. To explore predictive covariates, a mixed-effects single-covariate meta-regression was conducted. Missing covariate data were imputed using multiple imputation. The meta-regression included covariates such as follow-up duration, sample size, mean age, BMI, and therapy modality. Regression coefficients, standard errors, confidence intervals, and p-values were calculated, with p < 0.05 considered statistically significant. Outliers were identified in the forest plots, though no exclusions were made during the pooling process. Sensitivity analyses to assess the robustness of pooled estimates were not feasible due to the limited number of studies per subgroup. All analyses accounted for potential variability across studies, and findings were interpreted within the context of significant heterogeneity, publication bias, and imputed data considerations. Statistical and graphical outputs were prepared using R to ensure accuracy and reproducibility.

## 3 Results

**Fig. 1:**
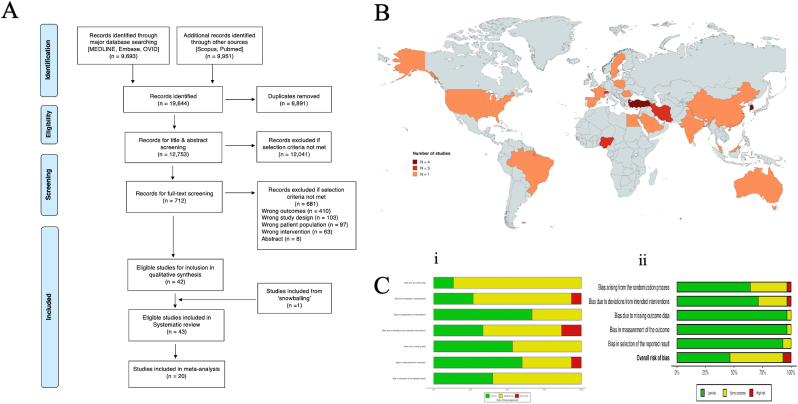
In Fig. 1A, the Preferred Reporting Items for Systematic Reviews and Meta-Analyses (PRISMA) flowchart outlining the study selection process is shown. In Fig. 1B, a world map indicates the origin of publications included in this study (n = 20). The countries are coloured according to whether n = 1, 2, 3, or 4 studies from these countries have been included in this systematic review. The legend at the bottom indicates the colour coding. The following countries are coloured: Austria (n = 1), Australia (n = 1), China (n = 3), Egypt (n = 1), France (n = 1), Iran (n = 4), Netherlands (n = 2), Nigeria (n = 2), Poland (n = 1), Romania (n = 1), Saudi Arabia (n = 1), South Korea (n = 1), Spain (n = 1), Sweden (n = 1), Switzerland (n = 3), Turkey (n = 3), and the United States of America (n = 2). In Fig. 1C (i), a risk of bias summary plot for randomized studies, using a bar chart of the distribution of risk-of-bias judgments for all included studies, is displayed across the domains of the Risk of Bias 2 (RoB-2) tool: Bias arising from the randomization process (Domain 1), Bias due to deviations from intended interventions (Domain 2), Bias due to missing outcome data (Domain 3), Bias in measurement of the outcome (Domain 4), Bias in selection of the reported result (Domain 5). In Fig. 1C (ii), a risk of bias summary plot for non-randomized studies is presented, using a bar chart of the distribution of risk-of-bias judgments for all included studies across the domains of the Risk of Bias in Non-Randomised Studies of Interventions (ROBINS-I) tool: Bias due to confounding (Domain 1), Bias in selection of participants (Domain 2), Bias in classification of interventions (Domain 3), Bias due to deviations from intended interventions (Domain 4), Bias due to missing data (Domain 5), Bias in measurement of outcomes (Domain 6), Bias in selection of the reported result (Domain 7). An overall risk-of-bias score, representing the collated judgments for all domains, is displayed at the bottom for each plot. Percentages (%) are depicted for clarity.

Of 19,644 records identified, 6891 duplicates were removed. 12,753 records were screened by title and abstract, of which 12,041 were excluded. The remaining 712 full-text articles were assessed for eligibility, and 670 were excluded with reasons (Fig. 1A), resulting in 42 being eligible for inclusion. An additional study was included through ‘snowballing’ of references (Luijsterburg et al., 2007). As such, a total of 43 studies were included in this systematic review (Fig. 1A). These studies encompassed a combined sample of 4129 patients. Out of these, 20 studies were included in the meta-analysis, with a combined sample size of 2187 patients, reflecting studies that met stricter criteria for quantitative pooling. The geographic distribution of study origins is depicted in Fig. 1B, with contributions from diverse regions, including North America, Europe, Asia, and the Middle East. The majority of studies originated from Turkey (n = 6), followed by Iran (n = 5) and the United States (n = 5).

The risk of bias was assessed using the ROBINS-I tool for non-randomized studies and the RoB 2 tool for RCTs. In the ROBINS-I tool, 1 study was classified as "serious" risk of bias and 1 study was rated as "critical" overall (Fig. 1C). Among the RCTs assessed using RoB 2, 13 studies showed "some concerns" and 2 studies demonstrated "high risk" of bias. Evidence levels, as determined using the Oxford Centre for Evidence-Based Medicine (OCEBM) criteria, classified the studies as level 1b (n = 14), level 2b (n = 26), and level 3b (n = 3). GRADE assessments categorized 39 studies as having "moderate" overall evidence quality, with 3 studies classified as "low" quality and 1 study classified as “high” quality. Detailed results of these evaluations are presented in Supplementary Table 4–7.

**Fig. 2:**
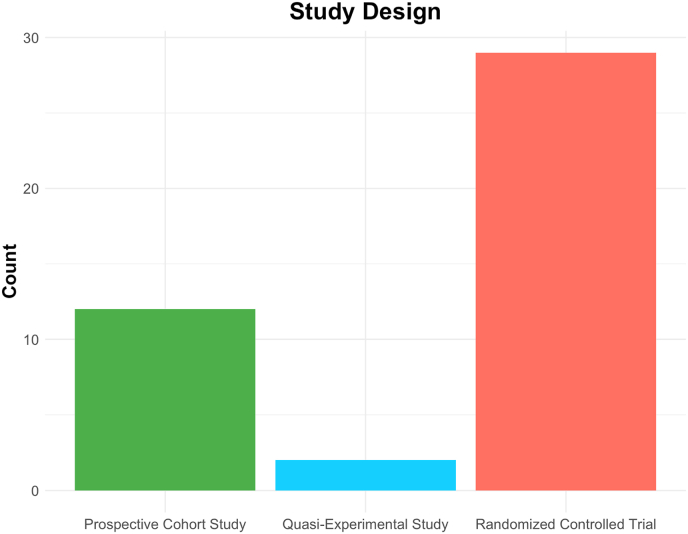
In Fig. 2, a bar plot visualizes the count of included studies by study design: prospective cohort studies (n = 12), quasi-experimental study (n = 2), and randomized controlled trials (RCTs) (n = 29). The study designs are further distinguished by colour.

**Fig. 3:**
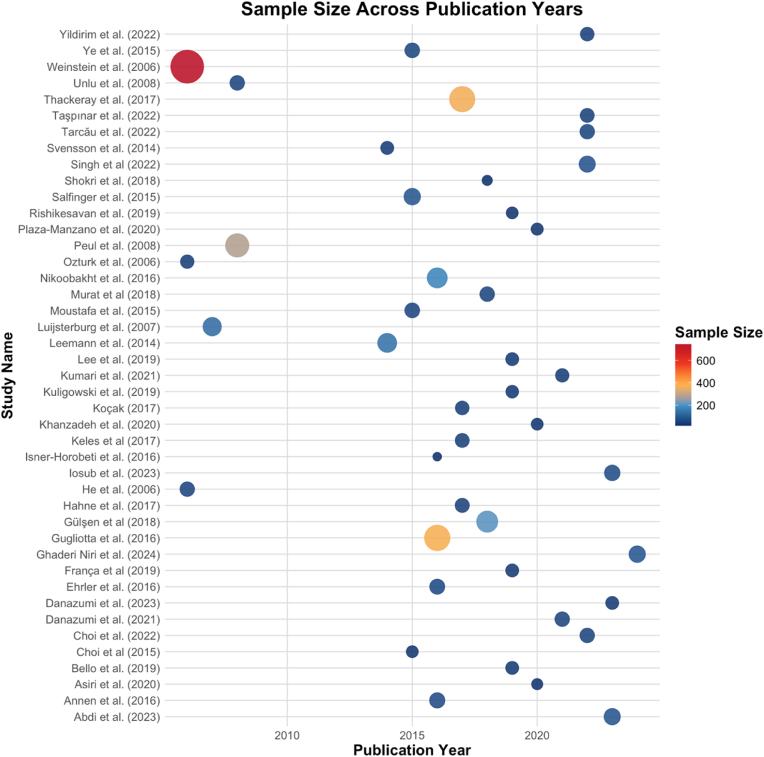
Fig. 3 illustrates the sample sizes of included studies stratified by publication year, with each point representing a study. The size and colour intensity of the points correspond to the study's sample size, ranging from small (blue) to large (red). Most studies are concentrated between 2015 and 2023, reflecting the recent focus on conservative therapies for lumbar disc herniation. Notably, Weinstein et al. (2006) is an outlier with the largest sample size.

Study characteristics, including country of origin, sample size, follow-up duration, and intervention type, are detailed in Table 1, while the distribution of study designs is visualized in Fig. 2. Of the included studies, 15 (48.4 %) investigated traction therapy, 4 (12.9 %) focused on physiotherapy, 5 (16.1 %) examined exercise-based interventions, 4 (12.9 %) explored manipulation therapy, and 4 (12.9 %) focused on mobilization. One study directly compared manipulation and mobilization, while the remaining 3 (9.7 %) were classified as miscellaneous, including novel interventions like Kinesio taping, electrotherapy, and individualized rehabilitation programs. The included studies consisted of randomized controlled trials (RCTs) (n = 28), prospective cohort studies (n = 12) and quasi-experimental studies (n = 2). Follow-up periods ranged from 4 weeks to over 24 months. Sample size and publication dates are visualized in Fig. 3. Sample sizes ranged from n = 17 to n = 753, and publication date from 2006 to 2023, with most studies having been published in the last decade.

## 4 Exercise therapy

Six studies encompassing 590 patients evaluated exercise-based interventions for lumbar disc herniation (LDH), exploring a range of modalities including Pilates, yoga, flexion- and extension-based exercises, and structured physiotherapy (Table 2). Across the studies, exercise therapy consistently demonstrated significant reductions in pain and disability, with improvements in functional and psychological outcomes. However, the effectiveness of specific exercises varied depending on patient characteristics, underlying pathology, and intervention timing.

### 4.1 Exercise-based intervention approaches

Exercise therapy for lumbar disc herniation (LDH) encompasses a variety of structured physical activities aimed at reducing pain, restoring function, and improving quality of life. The most common regimens include:

• *Core Stabilization Exercises*: Target the deep trunk muscles to improve spinal support and reduce mechanical strain.
• *McKenzie Therapy (Mechanical Diagnosis and Therapy, MDT):* A structured program emphasizing posture correction, repeated movements, and pain centralization, particularly effective for sciatica and discogenic pain.
• *Flexion- and Extension-Based Routines:* Designed to address specific biomechanical imbalances, with extension exercises often prescribed for posterior disc bulges to decompress neural structures.
• *Mind-Body Approaches*: Yoga and Pilates integrate controlled movements with neural mobilization and breathing to address flexibility, strength, and psychosocial factors.
• *Structured Physiotherapy Programs*: Combine supervised exercises with education and manual therapy to promote functional independence and reduce fear-avoidance behaviours.

### 4.2 Pain and disability reduction

All six studies reported substantial reductions in pain and disability, as measured by the Visual Analog Scale (VAS) and Oswestry Disability Index (ODI). Pilates-based core stabilization programs, as investigated by Taşpınar et al. (2022), achieved a 62.3 % reduction in VAS scores and a 41.5 % reduction in ODI (p < 0.001) (Taşpınar et al., 2023). Similarly, a 12-week yoga program, tailored to neuropathic pain patients, resulted in significant improvements in neuropathic pain scores (LANSS, p < 0.001), ODI (p < 0.001), and QoL measures, with sustained benefits observed at six months (Yildirim and Gultekin, 2022). Flexion- and extension-based exercises further reinforced the value of targeted programs, with Abdi et al. (2023) demonstrating superior pain (VAS, p < 0.001) and disability (ODI, p < 0.001) reductions in patients performing extension-based routines compared to flexion-based exercises (Abdi et al., 2023). These findings highlight the utility of extension-based programs in postoperative rehabilitation and suggest they may be more effective than flexion exercises for improving trunk extensor endurance and restoring lumbar lordosis.

### 4.3 Functional and psychosocial gains

Beyond pain relief, exercise therapy improved flexibility, muscle endurance, and psychosocial well-being. McKenzie therapy was particularly effective in centralizing radicular pain and enhancing lumbar mobility, while yoga uniquely addressed neuropathic pain and psychological stress, yielding moderate effect sizes for disability and pain outcomes (Cohen's d = 0.7). Structured physiotherapy programs provided sustained benefits in reducing the fear of mobilising against pain and improving return-to-work rates, as demonstrated by Svensson et al. (2014), with improvements maintained over 24 months (p < 0.001) (Svensson et al., 2014). These findings suggest that mind-body approaches like Pilates and Yoga may be particularly effective for patients with chronic and psychosocially complex cases, whereas structured physiotherapy offers long-term benefits across broader patient populations. One RCT with an economic evaluation reported that while physiotherapy plus GP care improved perceived recovery, it conferred no QALY benefit and was not cost-effective (ICER ≈ €6224 per additional recovered patient) (Luijsterburg et al., 2007).

### 4.4 Timing and tailored adjustments

The timing of intervention also influenced outcomes. Thackeray et al. (2017) found that early physiotherapy (within six weeks) yielded modest benefits for low back pain bothersomeness at one year (p = 0.05), although ODI and VAS outcomes were comparable to delayed physiotherapy (Thackeray et al., 2017). The benefits of timing were most pronounced in patients with severe initial symptoms or a preference for nonsurgical care. Moreover, the inclusion of patient-specific adjustments enhanced efficacy, as demonstrated by Asiri et al. (2020), who combined tailored three-dimensional lumbar traction (PS3DLT) with exercise therapy (Asiri et al., 2020). This approach reduced pain (VAS: 8.5 to 3.2, p < 0.001) and disability (ODI: 53.5 %–31.3 %, p < 0.001) through targeted decompression of affected spinal segments, highlighting the potential of precision adjustments for patients with localized nerve root compression.

#### 4.4.1 Summary

Key themes emerging from the studies include the importance of patient selection and the alignment of exercise modalities with clinical presentations. For chronic low back pain and psychosocially complex cases, mind-body programs like Pilates and yoga appear optimal, integrating neural mobilization and stabilization while addressing psychological contributors to pain. Postoperative patients benefited most from structured routines emphasizing extension-based movements, which restored lumbar lordosis and enhanced back muscle endurance, with evidence suggesting extension exercises may outperform flexion-based exercises in reducing disability and pain. Early physiotherapy showed limited additional benefits overall but remains critical for patients with acute symptom onset or high baseline disability. Precision approaches, such as PS3DLT, highlight the potential of tailored decompression in combination with exercise therapy, though broader applicability requires further exploration. Together, these findings underscore the need for personalized exercise regimens, adapted to patient-specific factors, to maximize the therapeutic benefits of exercise in LDH management.

## 5 Manipulation therapy

Eight studies evaluating spinal manipulation therapy (SMT) for lumbar disc herniation (LDH) with or without associated radiculopathy highlighted consistent benefits in pain reduction, disability improvement, and neural mobility (Table 3). However, the effectiveness varied based on patient characteristics, intervention protocols, and imaging findings, emphasizing the importance of tailored approaches.

### 5.1 Manipulation therapy approaches

• Spinal Manipulation Therapy (SMT) typically involves high-velocity, low-amplitude (HVLA) thrust techniques applied to specific spinal segments. This standard intervention aims to restore joint mobility, reduce mechanical dysfunction, alleviate pain, and improve overall functional outcomes. SMT is generally directed at spinal joints and surrounding soft tissues, often without directly addressing neural structures.
• Neurodynamic Mobilization (NM), on the other hand, targets the mobility and sensitivity of peripheral nerves. Techniques involve controlled limb movements designed to mobilize specific nerves while minimizing mechanical stress. This approach is particularly relevant in LDH cases with radiculopathy, where neural irritation or compression contributes to symptoms such as neuropathic pain and reduced range of motion. Unlike SMT, NM directly engages nerve-related dysfunctions, making it a complementary intervention.

### 5.2 Pain and disability reduction

Across all eight studies, SMT showed significant reductions in pain and disability, with notable differences based on herniation morphology, chronicity, and adjunctive therapies. In terms of combination approaches, Plaza-Manzano et al. found that combining SMT with NM enhanced outcomes in patients with LDH and radiculopathy (Plaza-Manzano et al., 2020). This approach yielded superior improvements in neuropathic symptoms (S-LANSS, p = 0.008) and straight leg raise (SLR, SMD = 1.05, p = 0.013) compared to SMT alone. Singh et al. demonstrated that spinal mobilization with leg movement (SMWLM), a specialized NM technique, outperformed HVLA thrusts and NM alone in reducing pain (VAS: 6.05 ± 1.32), disability (ODI: 15.65 ± 2.43), and improving SLR (15.06 ± 3.1°, p < 0.001). These findings highlight the added value of NM in addressing neural mobility and mechanical sensitivity. In terms of the standard approach, Ehrler et al. reported that SMT alone provided significantly greater leg pain reductions in patients with sequestrated herniations compared to extrusions at one month (mean reduction: 4.34 vs. 2.39 NRS points, p = 0.02) (Ehrler et al., 2016). Similarly, Leemann et al. showed that SMT led to substantial improvements across all time points, with 90.5 % of acute and 89.2 % of chronic patients reporting overall improvement at one year (p < 0.0001) (Leemann et al., 2014). These results emphasize that SMT remains highly effective as a standalone therapy, especially when anatomical factors such as herniation type are considered.

### 5.3 Neural mobility and functional outcomes

The integration of NM techniques into SMT protocols further improved neural mobility and functional outcomes. Danazumi et al. demonstrated that mobilization combined with NM resulted in better activity limitation, functional mobility, and quality of life outcomes at all follow-ups compared to SMT alone (p < 0.05) (Danazumi et al., 2023). Plaza-Manzano et al. similarly observed that NM enhanced SLR outcomes and reduced neuropathic symptoms, suggesting that addressing nerve-specific dysfunctions can optimize LDH management (Plaza-Manzano et al., 2020).

### 5.4 Imaging-guided approaches

Several studies highlighted the role of imaging findings in tailoring SMT interventions. Annen et al. found that Modic-positive patients experienced greater leg pain reductions (NRS, p = 0.02) and disability improvements (ODI, p = 0.012) at two weeks compared to Modic-negative patients (Annen et al., 2016). However, at one year, Modic Type II patients reported significantly better outcomes than Modic Type I, suggesting that imaging-based stratification may improve long-term efficacy. Ehrler et al. similarly reported that sequestration-type herniations responded better to SMT than extrusions, with significant differences in leg pain reduction at one month (p = 0.02) (Ehrler et al., 2016). Paracentral-plus-foraminal herniations also showed better back pain reduction compared to foraminal herniations alone (p = 0.04).

### 5.5 Chronicity and timing

The duration of symptoms influenced the speed and magnitude of recovery, with acute patients generally responding faster to SMT. Leemann et al. found that acute patients showed greater improvements at three months, while chronic patients demonstrated sustained benefits at one year (89.2 % improved, p < 0.0001) (Leemann et al., 2014). Hahne et al. reported that individualized functional restoration programs combined with SMT produced significant short-term gains in back pain but required longer durations to impact activity limitation (Hahne et al., 2017). Danazumi et al. highlighted the value of NM techniques in chronic cases, showing meaningful improvements in activity limitation, functional mobility, and quality of life outcomes at long-term follow-ups (p < 0.05) (Danazumi et al., 2023). These findings suggest that multimodal approaches and prolonged interventions are critical for chronic cases.

#### 5.5.1 Summary

Manipulation therapy effectively addressed pain, disability, and neural mobility in LDH patients, particularly when interventions were tailored to herniation morphology and chronicity. Spinal manipulation therapy (SMT) achieved robust improvements in acute and chronic patients, with neurodynamic mobilization (NM) further enhancing outcomes in radiculopathy cases. Studies highlighted imaging-based stratification's role, where Modic changes or herniation type guided therapy selection for superior pain reduction and functional restoration. Techniques like spinal mobilization with leg movement (SMWLM) outperformed standard SMT for neural mobility and symptom relief. Integrating NM with SMT proved particularly advantageous, emphasizing the value of multimodal approaches for optimizing patient outcomes, especially in cases with nerve-related dysfunction.

## 6 Traction therapy

Ten studies evaluated the efficacy of traction therapy for lumbar disc herniation (LDH), focusing on its impact on pain reduction, functional improvement, and anatomical changes (Table 4). These studies assessed various modalities, including patient-specific three-dimensional traction, nonsurgical spinal decompression, and adjunctive therapies like Kinesio taping and motor control training.

### 6.1 Traction therapy approaches

Traction therapy encompasses mechanical or device-assisted methods aimed at reducing intradiscal pressure, alleviating nerve compression, and enhancing spinal alignment. Common approaches include:

• Patient-Specific Three-Dimensional Lumbar Traction (PS3DLT): This technique uses customized mechanical devices to apply decompression forces in three planes (sagittal, coronal, and transverse). It targets the specific anatomical region of the herniation, optimizing decompression and providing relief by focusing on the unique characteristics of each patient's spinal structure.
• Nonsurgical Spinal Decompression Therapy (NSDT): This advanced mechanical therapy creates a negative pressure environment within the intervertebral discs, encouraging the retraction of herniated material and promoting nutrient flow to the disc. NSDT often uses computerized devices to control force and duration, enhancing precision compared to traditional methods.
• Continuous Lumbar Traction (CLT): A mechanical method that applies a steady pulling force along the spine. CLT aims to reduce pressure on nerve roots and alleviate pain by elongating the spine and creating space between vertebrae. It is less tailored than PS3DLT or NSDT but can be effective for general cases.
• Motor Control Training (MCT): Unlike passive traction, MCT focuses on active neuromuscular re-education. It strengthens core muscles and enhances the stability of the lumbar spine, reducing the mechanical stress on intervertebral discs. MCT is particularly useful for chronic conditions where weakness and instability exacerbate symptoms.
• Kinesio Taping (KT): KT involves applying elastic therapeutic tape to support and stabilize the lumbar region without restricting movement. It is thought to reduce pain, promote circulation, and enhance proprioception, complementing the effects of traction therapy.
• Vojta Therapy: A neurophysiological approach that activates reflexive muscle patterns through specific pressure points. When combined with traction, it can improve muscle coordination and enhance functional outcomes in LDH patients.

### 6.2 Pain and disability reduction

Traction therapy demonstrated consistent efficacy in reducing pain and improving function across diverse patient populations. Asiri et al. found that PS3DLT significantly reduced pain (VAS: 8.5 to 3.2, p < 0.001) and disability (ODI: 53.5 %–31.3 %, p < 0.001) over four weeks (Asiri et al., 2020). Similarly, Choi E et al. reported that NSDT achieved a 27.6 % reduction in the herniation index (HI) compared to 7.1 % with pseudodecompression (p = 0.017), alongside improved leg pain and functional scores (K-ODI, p < 0.05) (Choi et al., 2022). Moustafa et al. observed significant reductions in back (F = 33.6, p < 0.001) and leg pain (F = 67.2, p < 0.001) with lumbar extension traction (Moustafa and Diab, 2013), while He et al. showed that herbal magnetic corsets combined with traction therapy resulted in gradual pain relief (p < 0.05) and better functional improvement (p < 0.001) (He et al., 2006). Choi J et al. compared NSDT and continuous lumbar traction, finding significant reductions in VAS and ODI scores in both groups (p < 0.05) (Choi et al., 2015). However, no differences were observed between modalities, highlighting similar efficacy in pain and disability reduction. Franca et al. demonstrated that MCT outperformed transcutaneous electrical nerve stimulation (TENS) in reducing pain (mean difference = 3.3, p < 0.001) and disability (mean difference = 8.4, p < 0.001) (França et al., 2019). Gŭlşen et al. noted significant pain reduction across all treatment groups, including traction and home exercises (p < 0.05), but no added benefit of traction over other physiotherapy modalities (Gŭlşen et al., 2018). Iosub et al. showed that combining Vojta therapy with traction led to greater improvements in pain (VAS: p = 0.000) and disability (ODI: p = 0.000) compared to standard physical therapy alone (Iosub et al., 2023). Keles et al. found that KT reduced pain during activity (NRS, p < 0.05) and maintained functional gains (ODI, p < 0.001) at 12 weeks, while reducing reliance on analgesics compared to placebo (Keles et al., 2017).

### 6.3 Imaging-based outcomes

Traction therapy's impact on disc morphology was evident in several studies. Ozturk et al. reported a 23 % reduction in herniation size with continuous traction (p < 0.01), particularly in patients with larger herniations (Ozturk et al., 2006). Similarly, Choi E et al. found significant reductions in disc volume and herniation index with NSDT, correlating with functional improvements (Choi et al., 2022).

Moustafa et al. demonstrated enhanced intervertebral motion and restored lumbar lordosis with extension traction (p < 0.001) (Moustafa and Diab, 2013), while He et al. noted no significant imaging changes but observed functional improvements attributed to adjunctive corset use (He et al., 2006).

### 6.4 Adjunctive and combined therapies

Adjunctive therapies amplified traction outcomes by targeting core stability, neurophysiological activation, or inflammation. Franca et al. highlighted the superiority of MCT over TENS in improving core muscle activation (mean difference = 1.5, p < 0.001) and sensory pain quality (mean difference = 10.3, p < 0.001) (França et al., 2019). Keles et al. demonstrated KT's efficacy in reducing pain and maintaining functional gains, especially during activity (Keles et al., 2017). Iosub et al. noted that Vojta therapy enhanced mobility and strength (p < 0.001) when combined with standard physical therapy (Iosub et al., 2023).

### 6.5 Chronicity, timing, and personalization

Patient-specific factors influenced traction outcomes significantly. Asiri et al. emphasized the need for tailored traction parameters to achieve optimal decompression (Asiri et al., 2020). Gŭlşen et al. highlighted that traction may not offer additional benefits over exercise-based approaches in chronic cases (Gŭlşen et al., 2018), while Franca et al. demonstrated MCT's value in managing long-standing radiculopathy (França et al., 2019). Choi J et al. observed no significant differences between NSDT and continuous traction but suggested that longer treatment durations could yield more definitive results (Choi et al., 2015).

#### 6.5.1 Summary

Traction therapy demonstrated diverse benefits for LDH management, particularly when paired with adjunctive modalities or tailored to specific patient factors. Patient-specific three-dimensional lumbar traction (PS3DLT) and nonsurgical spinal decompression therapy (NSDT) excelled in reducing pain, disability, and disc herniation size, with PS3DLT showing significant advantages in precision-targeted decompression. Imaging studies confirmed disc volume reductions and restored spinal alignment, particularly in patients with larger herniations. Adjunctive therapies such as Kinesio taping (KT) and motor control training (MCT) enhanced outcomes by addressing core stability, functional gains, and pain sensitivity, while neurophysiological approaches like Vojta therapy showed complementary benefits in mobility and strength. Conversely, studies like Gŭlşen et al. highlighted that traction alone offered no superiority over exercise-based approaches in chronic cases (Gŭlşen et al., 2018). These findings emphasize traction's value as a component of multimodal care, requiring careful patient selection and personalization for maximum benefit.

## 7 Meta-analysis

**Fig. 4:**
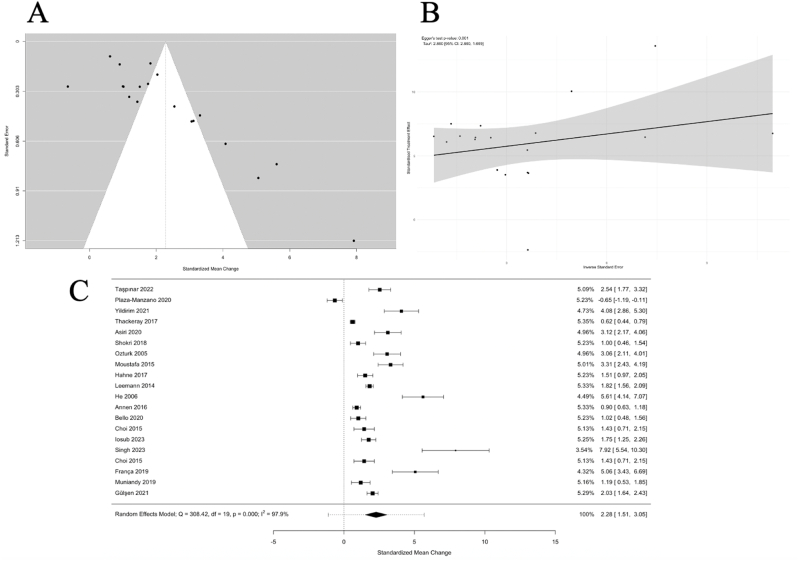
In Fig. 4A, a funnel plot visualizes the distribution of standardized mean change values (effect sizes) against the standard error for all studies included in the meta-analysis (n = 20). Each dot represents an individual study, and the funnel-shaped boundaries indicate the expected distribution in the absence of publication bias. Studies falling outside the boundaries suggest possible publication bias or heterogeneity. In Fig. 4B, an Egger's asymmetry plot is presented to statistically assess publication bias. The x-axis represents the inverse of the standard error, and the y-axis indicates the standardized mean change (effect size). The regression line and confidence interval (shaded area) demonstrate the relationship between these two parameters. Egger's test yielded p = 0.0031, indicating significant publication bias. Additionally, heterogeneity metrics such as I^2^ are reported in the upper corner of the graph. In Fig. 4C, a forest plot provides an overview of the pooled standardized mean change values with 95 % confidence intervals for each study (n = 20). The size of each data point reflects the weight of the study in the meta-analysis, with larger studies contributing more to the pooled estimate. The overall pooled effect size, represented by the diamond at the bottom, indicates a standardized mean change of 2.28 [1.51, 3.05]. Heterogeneity is significant, with I^2^ = 97.9 % and p < 0.0001. These findings highlight considerable variability in effect sizes across studies. **CI**: Confidence Interval; **SMC**: Standardized Mean Change; **I^2^**: Heterogeneity Index.

This meta-analysis synthesized results from 20 studies encompassing 2187 patients, evaluating the efficacy of conservative therapies for lumbar disc herniation (LDH) across three primary modalities: exercise therapy, manipulation therapy, and traction therapy. The overall pooled SMC was 2.28 (95 % CI: 1.51, 3.05), reflecting a large overall effect of conservative treatments on pain reduction and functional improvement (Fig. 4). When outcomes were examined separately, the pooled SMC for pain (VAS) across 15 studies was 2.48 (95 % CI: 1.56–3.39; *p* < 0.0001), while the pooled SMC for function (ODI) across 14 studies was 2.14 (95 % CI: 1.28–3.01; *p* < 0.0001). These findings confirm consistent improvements in both pain and disability with conservative therapy. However, heterogeneity was substantial (I^2^ = 97.9 %, p < 0.0001), warranting closer examination of the individual modalities and their variability (Fig. 4A and. B).

### 7.1 Publication bias

The funnel plot revealed an asymmetric distribution of effect sizes, indicating potential publication bias, which was confirmed by Egger's test (p = 0.0031). Studies with smaller sample sizes tended to report larger effect sizes (Fig. 4A and. B).

### 7.2 Comparative effectiveness of therapy modalities

**Fig. 5:**
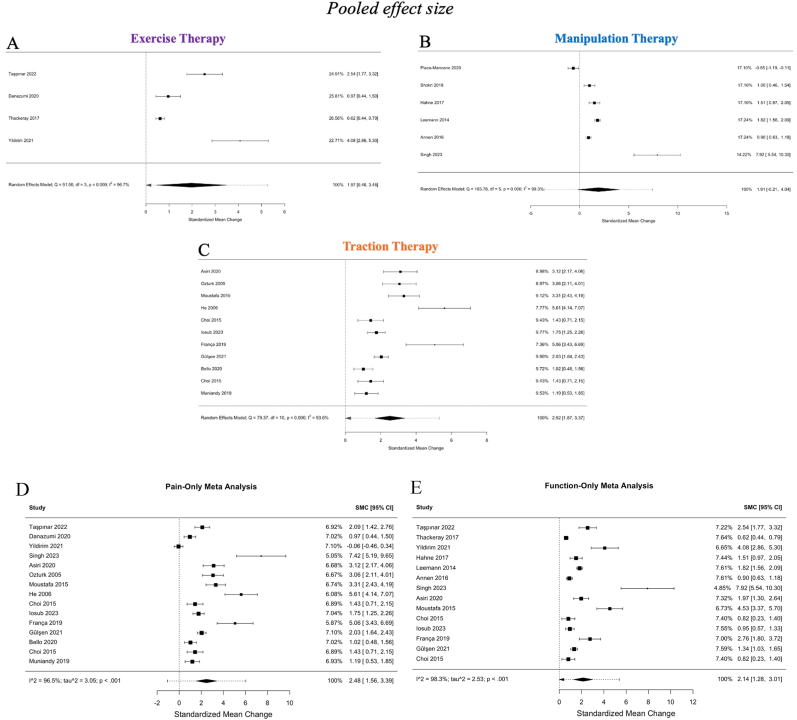
In Fig. 5A, a forest plot displays the pooled standardized mean change (SMC) values for exercise therapy in the management of lumbar disc herniation (n = 4 studies). Each study's effect size is shown alongside its 95 % confidence interval (CI). The random-effects model yielded a pooled SMC of 1.97 [0.46, 3.48], with significant heterogeneity observed (I^2^ = 56.7 %, p < 0.0001). The diamond at the bottom of the plot represents the pooled estimate, integrating the results of all included studies. In Fig. 5B, the forest plot summarizes the effects of manipulation therapy across six included studies. The individual and pooled SMC values are presented with their corresponding 95 % CIs. The pooled SMC is 1.91 [0.24, 4.04], with high heterogeneity (I^2^ = 99.3 %, p < 0.0001). Each study's contribution to the pooled effect size is visually indicated by the size of the corresponding data point. In Fig. 5C, the pooled results for traction therapy are shown, encompassing 10 included studies. The random-effects model yielded a pooled SMC of 2.52 [1.57, 3.37], indicating the effectiveness of this modality in improving outcomes for patients with lumbar disc herniation. Significant heterogeneity was also observed (I^2^ = 93.6 %, p < 0.0001). The forest plot visualizes the variability across studies and the overall effect. In Fig. 5D, the forest plot summarizes the effects of conservative therapy on pain (VAS) across 15 included studies. The individual and pooled standardized mean change (SMC) values are presented with their corresponding 95 % confidence intervals (CIs). The pooled SMC is 2.48 [1.56, 3.39], with very high heterogeneity (I^2^ = 96.5 %, p < 0.0001). In Fig. 5E, the forest plot summarizes the effects of conservative therapy on function (ODI) across 14 included studies. The individual and pooled SMC values are presented with their corresponding 95 % confidence intervals (CIs). The pooled SMC is 2.14 [1.28, 3.01], with very high heterogeneity (I^2^ = 98.3 %, p < 0.0001). **SMC**: Standardized Mean Change; **CI**: Confidence Interval; **I^2^**: Heterogeneity Index.

The pooled effect sizes differed substantially among the three primary modalities. Traction therapy exhibited the largest effect size (SMC = 2.52, 95 % CI: 1.57, 3.37), followed by exercise therapy (SMC = 1.97, 95 % CI: 0.46, 3.48) and manipulation therapy (SMC = 1.91, 95 % CI: 0.24, 4.04). However, it is important to note that significant heterogeneity was observed within each modality, with I^2^ values of 93.6 % for traction therapy, 99.3 % for manipulation therapy, and 56.7 % for exercise therapy. This highlights the variability in study protocols, populations, and outcome measures (Fig. 5A–. B, Fig. 5C).

### 7.3 Meta-regression results

**Fig. 6:**
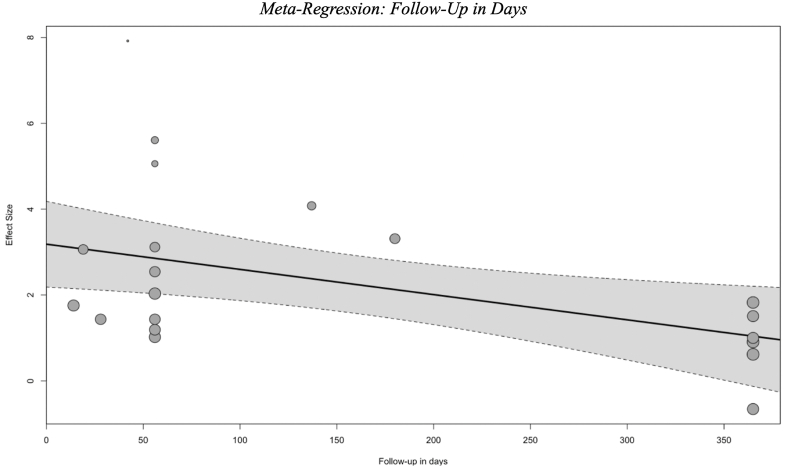
Fig. 6 presents the meta-regression analysis examining the relationship between follow-up duration (in days) and pooled effect size for conservative therapies in lumbar disc herniation. The regression line demonstrates a statistically significant inverse relationship (p < 0.001), indicating that shorter follow-up durations are associated with larger effect sizes. Shaded regions represent the 95 % confidence interval, reflecting the variability in effect size estimates. The analysis underscores the influence of follow-up duration as a critical factor in the observed heterogeneity, suggesting caution in interpreting studies with shorter follow-up periods.

The meta-regression analysis provided further insights into the potential sources of variability across studies (Table 5). The regression results revealed that follow-up duration significantly influenced effect sizes (estimate = −0.02, 95 % CI: −0.03, −0.01, p < 0.001), with shorter follow-up periods associated with larger observed effects (Fig. 6, Table 5).

**Table 5:** Summary results of the meta-regression results using an imputed dataset.

∼ *Co-Variate*	Estimate	Standard Error	95 % CI Lower	95 % CI Upper	*p*-value
*Sample Size*	0.01	0.01	−0.01	0.04	0.308
*Mean Age*	−0.02	0.13	−0.27	0.24	0.901
*Year of Publication*	−0.16	0.10	−0.35	0.03	0.102
*Manipulation Therapy*	3.48	2.19	−0.82	7.79	0.112
*Traction Therapy*	−1.87	1.42	−4.65	0.92	0.189
*Therapy Duration (Days)*	0.05	0.03	−0.01	0.11	0.116
*Follow-Up Duration (Days)*	−0.02	0.01	−0.03	−0.01	<0.001
*BMI*	0.60	0.34	−0.06	1.27	0.076
*Smoker*	0.03	0.12	−0.21	0.27	0.808
*Pain Duration (Months)*	0.05	0.06	−0.07	0.16	0.421

Table 5 presents the results of the meta-regression analysis using an imputed dataset. None of the patient- or study-level covariates achieved strong statistical significance, except for follow-up duration, which showed a small but significant negative association with the outcomes (estimate = −0.02, 95 % CI: −0.03 to −0.01, p < 0.001). This indicates that longer follow-up periods may be associated with slightly reduced effect sizes.

Neither sample size (p = 0.308) nor mean age of participants (p = 0.901) significantly predicted effect sizes, suggesting that the observed effects were not strongly influenced by these demographic or methodological factors. However, BMI showed a marginal association with outcomes (estimate = 0.60, 95 % CI: −0.06, 1.27, p = 0.076), indicating that patients with higher BMI may experience slightly greater benefits. The type of modality did not reach statistical significance.

### 7.4 Outliers and variability

Forest plots highlighted several outliers that contributed disproportionately to the observed heterogeneity (Fig. 5). For example, the study by Gülşen et al. (2021) reported a lower effect size for traction therapy compared to others, possibly due to differences in intervention protocols or patient populations (Gulsen et al., 2021). Similarly, Yildirim et al. (2021) reported an unusually high effect size for exercise therapy. Given the scarcity of studies for each modality, a sensitivity analysis was deemed not feasible (Yildirim and Gultekin, 2022).

#### 7.4.1 Summary

This meta-analysis underscores the effectiveness of conservative interventions for LDH, with traction therapy demonstrating the largest overall effect size. However, significant heterogeneity and potential publication bias highlight the need for tailored treatment strategies and further research to clarify the optimal application of these modalities. Meta-regression findings suggest that follow-up duration and individual patient factors may influence outcomes, emphasizing the importance of personalized approaches to maximize therapeutic benefits.

## 8 Discussion

This systematic review and meta-analysis included 43 studies with a total of 4043 patients in the qualitative synthesis, and 20 studies with 2187 patients were included in the meta-analysis. It represents the largest and most detailed evaluation of conservative physiotherapy modalities for lumbar disc herniation (LDH) to date, focusing on exercise therapy, manipulation therapy, and traction therapy. Our meta-analysis revealed that traction therapy had the highest pooled standardized mean change (SMC = 2.52, 95 % CI: 1.57, 3.37), followed by exercise therapy (SMC = 1.97, 95 % CI: 0.46, 3.48) and manipulation therapy (SMC = 1.91, 95 % CI: 0.24, 4.04). These results align with prior studies, such as the SPORT trial, demonstrating the effectiveness of conservative therapies in reducing pain and improving function, but our findings emphasize the substantial variability in outcomes across studies (I^2^ = 97.9 %). Importantly, the wide confidence intervals for exercise and manipulation therapies crossing the null, alongside the high RoB and lower certainty of evidence, suggest uncertainty in their true effects. As such, the pooled estimates should be interpreted as an overview of effects rather than an absolute measure of efficacy.

The heterogeneity observed in our analysis mirrors findings in previous studies. For example, Gülşen et al. (2021), which was included in our review, reported a significantly lower effect size for traction therapy compared to other studies, likely reflecting differences in intervention protocols or patient populations (Gulsen et al., 2021). Conversely, Yildirim et al. reported an unusually high effect size for exercise therapy, potentially due to the inclusion of chronic neuropathic pain patients, a subgroup known to benefit disproportionately from mind-body approaches such as yoga (Yildirim and Gultekin, 2022). These findings underscore the importance of tailoring therapy to specific patient characteristics, but our meta-analysis lacked sufficient data to quantitatively validate these subgroup-specific effects.

Our findings also highlight significant methodological gaps in the current literature. Follow-up duration emerged as a critical predictor of variability in effect sizes (p < 0.001), with shorter follow-ups associated with larger treatment effects. This trend is consistent with prior meta-analyses on conservative therapies, which have noted that short-term improvements may overestimate therapeutic benefits due to the natural resolution of LDH symptoms over time. For example, Svensson et al. (2014) observed sustained benefits of structured physiotherapy programs at 24 months, emphasizing the need for long-term follow-up in future studies to disentangle true therapeutic effects from natural recovery (Svensson et al., 2014). Publication bias was evident in our analysis, with smaller studies reporting disproportionately larger effect sizes, as confirmed by Egger's test (p = 0.0031). This issue, frequently noted in LDH research, emphasizes the need for more high-quality, large-scale studies to validate findings and reduce the influence of selective reporting.

While traction therapy showed the highest pooled effect size in this review, heterogeneity in its intervention protocols complicates interpretation. For instance, Asiri et al. (2020) found that patient-specific three-dimensional lumbar traction (PS3DLT) was more effective than standard continuous traction, suggesting that precision-targeted approaches may enhance outcomes (Asiri et al., 2020). However, the limited availability of comparative studies prevents definitive conclusions about the relative efficacy of different traction modalities or how they compare to exercise and manipulation therapy.

This review also highlights the limited evidence on how different therapies perform across specific patient subgroups. While qualitative findings suggest that chronic pain patients may benefit more from mind-body approaches like yoga, and postoperative patients may respond better to extension-based exercise routines, these insights remain speculative without direct quantitative comparisons. Future research should address this gap by conducting head-to-head randomized controlled trials with standardized protocols and outcome measures, focusing on subgroup-specific effects. Advanced techniques, such as machine learning, could also help identify predictors of treatment success and guide personalized therapy recommendations.

While this review provides a comprehensive synthesis of conservative therapies for LDH, several limitations must be acknowledged. The overall certainty of evidence was predominantly moderate, meaning confidence in the effect estimates is limited, particularly given the risk of bias in several studies. Substantial heterogeneity, variable intervention protocols, and inconsistent outcome reporting further constrain robustness. Use of standardized mean change as the effect size enabled pooling across diverse measures but assumes comparability across scales, which may reduce clinical interpretability. Pooling across heterogeneous populations and therapies may also obscure subgroup effects. Although random-effects models were used, results remain sensitive to outliers, small-study effects, and evident publication bias, which may overstate efficacy. Finally, while meta-regression identified follow-up duration as a significant moderator, clinically important factors such as herniation morphology and neurological deficits could not be evaluated due to incomplete reporting. This review was not pre-registered on PROSPERO, which may increase the risk of reporting bias and limits methodological transparency.

## 9 Conclusion

This systematic review and meta-analysis shows that conservative therapies may be effective for managing lumbar disc herniation, improving both pain and function. Among the investigated modalities, traction therapy was associated with the largest pooled effect size in this analysis. However, due to high heterogeneity, a lack of head-to-head trials, wide confidence intervals for exercise and manipulation therapies, and variations in traction protocols, this finding does not constitute strong evidence for the superiority of one modality over another. These findings should be interpreted with caution, as the overall certainty of evidence was moderate and several studies carried notable risk of bias, limiting the strength of firm recommendations. Future high-quality, long-term, and comparative trials are needed to establish clear, evidence-based guidance and support personalized treatment strategies.

## Funding

None.

## Declaration of competing interest

The authors declare that they have no known competing financial interests or personal relationships that could have appeared to influence the work reported in this paper.

## References

[bib1] Abdi A., Bagheri S.R., Shekarbeigi Z., Usefvand S., Alimohammadi E. (2023). The effect of repeated flexion-based exercises versus extension-based exercises on the clinical outcomes of patients with lumbar disk herniation surgery: a randomized clinical trial. Neurol. Res..

[bib2] Al Qaraghli M.I., De Jesus O. (2025).

[bib3] Annen M., Peterson C., Leemann S., Schmid C., Anklin B., Humphreys B.K. (2016). Comparison of outcomes in MRI confirmed lumbar disc herniation patients with and without modic changes treated with high velocity, low amplitude spinal manipulation. J. Manip. Physiol. Ther..

[bib4] Asiri F., Tedla J.S., Ms D.A., Ahmed I., Reddy R.S., Gular K. (2020). Effects of patient-specific three-dimensional lumbar traction on pain and functional disability in patients with lumbar intervertebral disc prolapse. Niger. J. Clin. Pract..

[bib5] Bello B., Danazumi M.S., Kaka B. (2019). Comparative effectiveness of 2 manual therapy techniques in the management of lumbar radiculopathy: a randomized clinical trial. J Chiropr Med.

[bib6] Choi J., Lee S., Hwangbo G. (2015). Influences of spinal decompression therapy and general traction therapy on the pain, disability, and straight leg raising of patients with intervertebral disc herniation. J. Phys. Ther. Sci..

[bib7] Choi E., Gil H.Y., Ju J., Han W.K., Nahm F.S., Lee P.B. (2022). Effect of nonsurgical spinal decompression on intensity of pain and herniated disc volume in subacute lumbar herniated disc. Int. J. Clin. Pract..

[bib8] Cook E., Scantlebury A., Booth A., Turner E., Ranganathan A., Khan A. (2021). Surgery versus conservative management of stable thoracolumbar fracture: the PRESTO feasibility RCT. Health Technol. Assess..

[bib9] Corp I. (2021).

[bib10] Danazumi M.S., Bello B., Yakasai A.M., Kaka B. (2021). Two manual therapy techniques for management of lumbar radiculopathy: a randomized clinical trial. J. Osteopath. Med..

[bib11] Danazumi M.S., Nuhu J.M., Ibrahim S.U., Falke M.A., Rufai S.A., Abdu U.G. (2023). Effects of spinal manipulation or mobilization as an adjunct to neurodynamic mobilization for lumbar disc herniation with radiculopathy: a randomized clinical trial. J. Man. Manip. Ther..

[bib12] Deyo R.A., Cherkin D.C., Weinstein J., Howe J., Ciol M., Mulley A.G. (2000). Involving patients in clinical decisions: impact of an interactive video program on use of back surgery. Med. Care.

[bib13] Durieux N., Vandenput S., Pasleau F. (2013). OCEBM levels of evidence system. Rev. Med. Liege.

[bib14] Ehrler M., Peterson C., Leemann S., Schmid C., Anklin B., Humphreys B.K. (2016). Symptomatic, MRI confirmed, lumbar disc herniations: a comparison of outcomes depending on the type and anatomical axial location of the hernia in patients treated with high-velocity, low-amplitude spinal manipulation. J. Manip. Physiol. Ther..

[bib15] Fjeld O.R., Grøvle L., Helgeland J., Småstuen M.C., Solberg T.K., Zwart J.A. (2019). Complications, reoperations, readmissions, and length of hospital stay in 34 639 surgical cases of lumbar disc herniation. Bone Joint Lett. J.

[bib16] França F.J.R., Callegari B., Ramos L.A.V., Burke T.N., Magalhães M.O., Comachio J. (2019). Motor control training compared with transcutaneous electrical nerve stimulation in patients with disc herniation with associated radiculopathy: a randomized controlled trial. Am. J. Phys. Med. Rehabil..

[bib17] Ghaderi Niri H., Ghanavati T., Mostafaee N., Salahzadeh Z., Divandari A., Adigozali H. (2024). Oswestry disability index, roland-morris disability questionnaire, and Quebec back pain disability scale: Responsiveness and minimal clinically important changes in Iranian people with lumbar disc herniation following physiotherapy. Arch Bone Jt Surg.

[bib18] Gugliotta M., da Costa B.R., Dabis E., Theiler R., Jüni P., Reichenbach S. (2016). Surgical versus conservative treatment for lumbar disc herniation: a prospective cohort study. BMJ Open.

[bib19] Gŭlşen M., Atici E., Aytar A., Sahin F.N. (2018). Effects of traction therapy in addition to conventional physiotherapy modalities on pain and functionality in patients with lumbar disc herniation: randomized controlled study. Acta Med..

[bib20] Gulsen M., Atıcı E., Aytar A., Fatma N., Şahin F.N. (2021).

[bib21] Guyatt G.H., Oxman A.D., Vist G.E., Kunz R., Falck-Ytter Y., Alonso-Coello P. (2008). GRADE: an emerging consensus on rating quality of evidence and strength of recommendations. Bmj.

[bib22] Hahne A.J., Ford J.J., Hinman R.S., Richards M.C., Surkitt L.D., Chan A.Y. (2017). Individualized functional restoration as an adjunct to advice for lumbar disc herniation with associated radiculopathy. A preplanned subgroup analysis of a randomized controlled trial. Spine J..

[bib23] Hansson E., Hansson T. (2007). The cost-utility of lumbar disc herniation surgery. Eur. Spine J..

[bib24] He C., Chen P., Wang X., Ding M., Lan Q., Han M. (2006). The clinical effect of herbal magnetic corsets on lumbar disc herniation. Clin. Rehabil..

[bib25] Iosub M.E., Ianc D., Sîrbu E., Ciobanu D., Lazăr L. (2023). Vojta therapy and conservative physical therapy versus physical therapy only for lumbar disc protrusion: a comparative cohort study from Romania. Applied Sciences [Internet].

[bib26] Isner-Horobeti M.E., Dufour S.P., Schaeffer M., Sauleau E., Vautravers P., Lecocq J. (2016). High-force versus low-force lumbar traction in acute lumbar sciatica due to disc herniation: a preliminary randomized trial. J. Manip. Physiol. Ther..

[bib27] Katz J.N. (2006). Lumbar disc disorders and low-back pain: socioeconomic factors and consequences. J Bone Joint Surg Am.

[bib28] Keles B.Y., Yalcinkaya E.Y., Gunduz B., Bardak A.N., Erhan B. (2017). Kinesio taping in patients with lumbar disc herniation: a randomised, controlled, double-blind study. J. Back Musculoskelet. Rehabil..

[bib29] Khanzadeh R., Mahdavinejad R., Borhani A. (2020). The effect of suspension and conventional core stability exercises on characteristics of intervertebral disc and chronic pain in office staff due to lumbar herniated disc. Arch Bone Jt Surg.

[bib30] Koçak F.A., Tunç H., Sütbeyaz S.T., Akkuş S., Köseoğlu B.F., Yılmaz E. (2017). Comparison of the short-term effects of the conventional motorized traction with non-surgical spinal decompression performed with a DRX9000 device on pain, functionality, depression, and quality of life in patients with low back pain associated with lumbar disc herniation: a single-blind randomized-controlled trial. Turkish journal of physical medicine and rehabilitation.

[bib31] Kuligowski T., Dębiec-Bąk A., Skrzek A. (2019). Effectiveness of traction in young patients representing different stages of degenerative disc disease. Ortop. Traumatol. Rehabil..

[bib32] Kumari A., Quddus N., Meena P.R., Alghadir A.H., Khan M. (2021). Effects of one-fifth, one-third, and one-half of the bodyweight lumbar traction on the straight leg raise test and pain in prolapsed intervertebral disc patients: a randomized controlled trial. BioMed Res. Int..

[bib33] Lee C.-H., Heo S.J., Park S.H., Jeong H.S., Kim S.-Y. (2020). Functional changes in patients and morphological changes in the lumbar intervertebral disc after applying lordotic curve-controlled traction: a double-blind randomized controlled study. Medicina.

[bib34] Leemann S., Peterson C.K., Schmid C., Anklin B., Humphreys B.K. (2014). Outcomes of acute and chronic patients with magnetic resonance imaging-confirmed symptomatic lumbar disc herniations receiving high-velocity, low-amplitude, spinal manipulative therapy: a prospective observational cohort study with one-year follow-up. J. Manip. Physiol. Ther..

[bib35] Luijsterburg P.A., Lamers L.M., Verhagen A.P., Ostelo R.W., van den Hoogen H.J., Peul W.C. (2007). Cost-effectiveness of physical therapy and general practitioner care for sciatica. Spine (Phila Pa 1976).

[bib36] Moustafa I.M., Diab A.A. (2013). Extension traction treatment for patients with discogenic lumbosacral radiculopathy: a randomized controlled trial. Clin. Rehabil..

[bib37] Murat S., Uzunca K., Erden N. (2018). The effect of lumbar traction with two different load on clinic and functional status of patients with subacute lumbar disc herniation. Medeniyet Medical Journal.

[bib38] Nikoobakht M., Yekanineajd M.S., Pakpour A.H., Gerszten P.C., Kasch R. (2016). Plasma disc decompression compared to physiotherapy for symptomatic contained lumbar disc herniation: a prospective randomized controlled trial. Neurol. Neurochir. Pol..

[bib39] Oertel J., Sharif S., Zygourakis C., Sippl C. (2024). Acute low back pain: epidemiology, etiology, and prevention: WFNS spine committee recommendations. World Neurosurg. X.

[bib40] Ozturk B., Gunduz O.H., Ozoran K., Bostanoglu S. (2006). Effect of continuous lumbar traction on the size of herniated disc material in lumbar disc herniation. Rheumatol. Int..

[bib41] Peul W.C., van den Hout W.B., Brand R., Thomeer R.T., Koes B.W. (2008). Prolonged conservative care versus early surgery in patients with sciatica caused by lumbar disc herniation: two year results of a randomised controlled trial. Bmj.

[bib42] Plaza-Manzano G., Cancela-Cilleruelo I., Fernández-de-Las-Peñas C., Cleland J.A., Arias-Buría J.L., Thoomes-de-Graaf M. (2020). Effects of adding a neurodynamic mobilization to motor control training in patients with lumbar radiculopathy due to disc herniation: a randomized clinical trial. Am. J. Phys. Med. Rehabil..

[bib43] Pojskic M., Bisson E., Oertel J., Takami T., Zygourakis C., Costa F. (2024). Lumbar disc herniation: epidemiology, clinical and radiologic diagnosis WFNS spine committee recommendations. World Neurosurg. X.

[bib44] Salfinger H., Salomonowitz G., Friedrich K.M., Hahne J., Holzapfel J., Friedrich M. (2015). Nuclear magnetic resonance therapy in lumbar disc herniation with lumbar radicular syndrome: effects of the intervention on pain intensity, health-related quality of life, disease-related disability, consumption of pain medication, duration of sick leave and MRI analysis. Eur. Spine J..

[bib45] Sehat M., Tabaraii R., Lotfi S., Etebari M., Cheraghi M., Ahmadi A. (2023). Evaluation of surgical complications after herniated spinal lumbar disc surgery. Interdisciplinary Neurosurgery.

[bib46] Shokri E., Kamali F., Sinaei E., Ghafarinejad F. (2018). Spinal manipulation in the treatment of patients with MRI-Confirmed lumbar disc herniation and sacroiliac joint hypomobility: a quasi-experimental study. Chiropr. Man. Ther..

[bib47] Singh K. (2019).

[bib48] Singh V., Malik M. (2022). Effect of manual therapy on pain, disability and neural mobility in patients of lumbar prolapsed intervertebral disc: a randomized controlled trial. Adv. Rehabil..

[bib49] Sterne J.A., Hernán M.A., Reeves B.C., Savović J., Berkman N.D., Viswanathan M. (2016). ROBINS-I: a tool for assessing risk of bias in non-randomised studies of interventions. Bmj.

[bib50] Sterne J.A.C., Savović J., Page M.J., Elbers R.G., Blencowe N.S., Boutron I. (2019). RoB 2: a revised tool for assessing risk of bias in randomised trials. Bmj.

[bib51] Svensson G.L., Wendt G.K., Thomeé R. (2014). A structured physiotherapy treatment model can provide rapid relief to patients who qualify for lumbar disc surgery: a prospective cohort study. J. Rehabil. Med..

[bib52] Tarcău E., Ianc D., Sirbu E., Ciobanu D., Boca I.C., Marcu F. (2022). Effects of complex rehabilitation program on reducing pain and disability in patients with lumbar disc Protrusion-Is early intervention the best recommendation?. J. Personalized Med..

[bib53] Taşpınar G., Angın E., Oksüz S. (2023). The effects of pilates on pain, functionality, quality of life, flexibility and endurance in lumbar disc herniation. J Comp Eff Res.

[bib54] Team RC R: a language and environment for statistical computing. https://www.R-project.org.

[bib55] Thackeray A., Fritz J.M., Lurie J.D., Zhao W., Weinstein J.N. (2017). Nonsurgical treatment choices by individuals with lumbar intervertebral disc herniation in the United States: associations with long-term outcomes. Am. J. Phys. Med. Rehabil..

[bib56] Unlu Z., Tasci S., Tarhan S., Pabuscu Y., Islak S. (2008). Comparison of 3 physical therapy modalities for acute pain in lumbar disc herniation measured by clinical evaluation and magnetic resonance imaging. J. Manip. Physiol. Ther..

[bib57] Weinstein J.N., Tosteson T.D., Lurie J.D., Tosteson A.N., Hanscom B., Skinner J.S. (2006). Surgical vs nonoperative treatment for lumbar disk herniation: the spine patient outcomes research trial (SPORT): a randomized trial. JAMA.

[bib58] Wong T., Patel A., Golub D., Kirnaz S., Goldberg J.L., Sommer F. (2023). Prevalence of long-term low back pain after symptomatic lumbar disc herniation. World Neurosurg..

[bib59] Yang S., Kim W., Kong H.H., Do K.H., Choi K.H. (2020). Epidural steroid injection versus conservative treatment for patients with Lumbosacral radicular pain: a meta-analysis of randomized controlled trials. Medicine (Baltim.).

[bib60] Ye C., Ren J., Zhang J., Wang C., Liu Z., Li F. (2015). Comparison of lumbar spine stabilization exercise versus general exercise in young Male patients with lumbar disc herniation after 1 year of follow-up. Int. J. Clin. Exp. Med..

[bib61] Yildirim P., Gultekin A. (2022). The effect of a stretch and strength-based yoga exercise program on patients with neuropathic pain due to lumbar disc herniation. Spine (Phila Pa 1976).

