# Supplementary material for: Exercise, manipulation and traction physiotherapy in the conservative management of lumbar disc herniation: A systematic review and meta-analysis
Source: Brain Spine. 2025 Oct 16;5:105632. doi: 10.1016/j.bas.2025.105632 (PMC12595123; doi:10.1016/j.bas.2025.105632)
Supplement: Multimedia component 1 [file mmc1.docx]

**SUPPLEMENTAL DIGITAL CONTENT 1**

**Exercise, Manipulation and Traction Physiotherapy in the Conservative Management of Lumbar Disc Herniation: A Systematic Review and Meta-Analysis.**

Santhosh G. Thavarajasingam^1,5,6*^

Daniele S.C. Ramsay^1,2^ Srikar R. Namireddy^1,2^ ; Abith G Kamath^1.2^

Sree Kanakala^1,2^  ; Hasan Zaidi^1,2^ ; Rishi Parikh^1,2^; Amaan Peerbhai^1,2^

Hariharan Subbiah Ponniah^1,2^ ; Aksaan Arif^1,2^ ;Ahmed Salih^1,2^

Ahkash Thavarajasingam^1,3^ ; Daniel Scurtu^1^

Dragan Jankovic^5^; Andreas Kramer^5,6^

Florian Ringel^5,6^

**INSTITUTION:**

1. Imperial Brain & Spine Initiative, Imperial College London, London, United Kingdom
2. Faculty of Medicine, Imperial College London, London, United Kingdom.
3. Faculty of Medicine, Medizinische Hochschule Hannover, Hannover, Germany.
4. Center for Spinal Surgery and Neurotraumatology, Berufsgenossenschaftliche Unfallklinik Frankfurt am Main, Germany
5. Department of Neurosurgery, LMU Ludwig-Maximilians-University Hospital Munich, Munich, Germany
6. EANS Spine Section, European Association of Neurosurgical Societies, Europe.

Table of Contents

[Supplementary Table 1: Search strategy. 3](#_Toc183245264)

[Supplementary Table 2: Inclusion and exclusion criteria. 7](#_Toc183245265)

[Supplementary Table 3: Table of extracted variables. 8](#_Toc183245266)

[Supplementary Table 4: Risk of bias analysis of all randomised studies (ROBINS-I tool). 11](#_Toc183245267)

[Supplementary Table 5: Risk of bias analysis of all randomised studies (ROB-2 tool). 13](#_Toc183245268)

[Supplementary Table 6: Level of evidence based on the Oxford Centre of Evidence-Based Medicine (OCEBM) Levels of Evidence. 15](#_Toc183245269)

[Supplementary Table 7: GRADE (Grading of Recommendations, Assessment, Development and Evaluations) scoring for all studies. 17](#_Toc183245270)

#

# **Supplementary Table 1:** Search strategy.

| **Database** | **Search terms** | **Publication dates** | **Results (n)** |
| --- | --- | --- | --- |
| Medline | ((("lumbar disc herniation".sh. OR "lumbar disc displacement".sh. OR "intervertebral disc displacement".sh. OR "lumbar herniation".tw. OR "lumbar disc disease".tw. OR "herniated disc".tw. OR "slipped disc".tw. OR "disc prolapse".tw. OR "disc protrusion".tw. OR "disc extrusion".tw. OR "disc sequestration".tw. OR "disc rupture".tw. OR "disc derangement".tw. OR "nucleus pulposus herniation".tw. OR "nucleus pulposus prolapse".tw. OR "disc displacement".tw. OR "intervertebral disc herniation".tw. OR "lumbar disc pathology".tw.) AND (("physical therapy".sh. OR "physical therapy modalities".sh. OR "exercise therapy".sh. OR "manual therapy".sh. OR "chiropractic".sh. OR "spinal manipulation".sh. OR "acupuncture".sh. OR "massage therapy".sh. OR "therapeutic exercise".sh. OR "traction".sh. OR "heat therapy".sh. OR "cold therapy".sh. OR "electrotherapy".sh. OR "injection therapy".sh. OR "steroid injections".sh. OR "epidural injections".sh. OR "transforaminal injections".sh. OR "facet joint injections".sh. OR "laser therapy".sh. OR "laser nucleoplasty".sh. OR "yoga".sh. OR "Pilates".sh. OR "physiotherapy".sh. OR "non-surgical treatment".tw. OR "conservative treatment".tw.) OR ("core strengthening".tw. OR "core stability".tw. OR "stretching exercises".tw. OR "aerobic exercise".tw. OR "mobilization".tw. OR "osteopathy".tw. OR "neural mobilization".tw. OR "ergonomic education".tw. OR "posture education".tw. OR "transcutaneous electrical nerve stimulation".tw. OR "TENS".tw. OR "dry needling".tw. OR "back school".tw. OR "mechanical traction".tw. OR "postural training".tw.)))) | 1948 - 2024 | n = 8,359 |
| Embase | ((("lumbar disc herniation".sh. OR "lumbar disc displacement".sh. OR "intervertebral disc displacement".sh. OR "lumbar herniation".tw. OR "lumbar disc disease".tw. OR "herniated disc".tw. OR "slipped disc".tw. OR "disc prolapse".tw. OR "disc protrusion".tw. OR "disc extrusion".tw. OR "disc sequestration".tw. OR "disc rupture".tw. OR "disc derangement".tw. OR "nucleus pulposus herniation".tw. OR "nucleus pulposus prolapse".tw. OR "disc displacement".tw. OR "intervertebral disc herniation".tw. OR "lumbar disc pathology".tw.) AND (("physical therapy".sh. OR "physical therapy modalities".sh. OR "exercise therapy".sh. OR "manual therapy".sh. OR "chiropractic".sh. OR "spinal manipulation".sh. OR "acupuncture".sh. OR "massage therapy".sh. OR "therapeutic exercise".sh. OR "traction".sh. OR "heat therapy".sh. OR "cold therapy".sh. OR "electrotherapy".sh. OR "injection therapy".sh. OR "steroid injections".sh. OR "epidural injections".sh. OR "transforaminal injections".sh. OR "facet joint injections".sh. OR "laser therapy".sh. OR "laser nucleoplasty".sh. OR "yoga".sh. OR "Pilates".sh. OR "physiotherapy".sh. OR "non-surgical treatment".tw. OR "conservative treatment".tw.) OR ("core strengthening".tw. OR "core stability".tw. OR "stretching exercises".tw. OR "aerobic exercise".tw. OR "mobilization".tw. OR "osteopathy".tw. OR "neural mobilization".tw. OR "ergonomic education".tw. OR "posture education".tw. OR "transcutaneous electrical nerve stimulation".tw. OR "TENS".tw. OR "dry needling".tw. OR "back school".tw. OR "mechanical traction".tw. OR "postural training".tw.)))) | 1947 - 2024 | n = 4,870 |
| Scopus | TITLE-ABS-KEY ( ( ( "lumbar disc herniation" OR "lumbar disc displacement" OR "intervertebral disc displacement" OR "lumbar herniation" OR "lumbar disc disease" OR "herniated disc" OR "slipped disc" OR "disc prolapse" OR "disc protrusion" OR "disc extrusion" OR "disc sequestration" OR "disc rupture" OR "disc derangement" OR "nucleus pulposus herniation" OR "nucleus pulposus prolapse" OR "disc displacement" OR "intervertebral disc herniation" OR "lumbar disc pathology" ) AND ( ( "physical therapy" OR "physical therapy modalities" OR "exercise therapy" OR "manual therapy" OR "chiropractic" OR "spinal manipulation" OR "acupuncture" OR "massage therapy" OR "therapeutic exercise" OR "traction" OR "heat therapy" OR "cold therapy" OR "electrotherapy" OR "injection therapy" OR "steroid injections" OR "epidural injections" OR "transforaminal injections" OR "facet joint injections" OR "laser therapy" OR "laser nucleoplasty" OR "yoga" OR "Pilates" OR "physiotherapy" OR "non-surgical treatment" OR "conservative treatment" ) OR ( "core strengthening" OR "core stability" OR "stretching exercises" OR "aerobic exercise" OR "mobilization" OR "osteopathy" OR "neural mobilization" OR "ergonomic education" OR "posture education" OR "transcutaneous electrical nerve stimulation" OR "TENS" OR "dry needling" OR "back school" OR "mechanical traction" OR "postural training" ) ) ) ) | 1943 - 2024 | n = 6,190 |
| Pubmed | ((("lumbar disc herniation"[tiab] OR "lumbar disc displacement"[tiab] OR "intervertebral disc displacement"[MeSH] OR "lumbar herniation"[tiab] OR "lumbar disc disease"[tiab] OR "herniated disc"[tiab] OR "slipped disc"[tiab] OR "disc prolapse"[tiab] OR "disc protrusion"[tiab] OR "disc extrusion"[tiab] OR "disc sequestration"[tiab] OR "disc rupture"[tiab] OR "disc derangement"[tiab] OR "nucleus pulposus herniation"[tiab] OR "nucleus pulposus prolapse"[tiab] OR "disc displacement"[tiab] OR "intervertebral disc herniation"[tiab] OR "lumbar disc pathology"[tiab])) AND (("physical therapy"[tiab] OR "physical therapy modalities"[MeSH] OR "exercise therapy"[MeSH] OR "manual therapy"[tiab] OR "chiropractic"[MeSH] OR "spinal manipulation"[tiab] OR "acupuncture"[MeSH] OR "massage therapy"[tiab] OR "therapeutic exercise"[tiab] OR "traction"[MeSH] OR "heat therapy"[tiab] OR "cold therapy"[tiab] OR "electrotherapy"[tiab] OR "injection therapy"[tiab] OR "steroid injections"[tiab] OR "epidural injections"[tiab] OR "transforaminal injections"[tiab] OR "facet joint injections"[tiab] OR "laser therapy"[MeSH] OR "laser nucleoplasty"[tiab] OR "yoga"[MeSH] OR "Pilates"[tiab] OR "physiotherapy"[tiab] OR "non-surgical treatment"[tiab] OR "conservative treatment"[tiab]) OR ("core strengthening"[tiab] OR "core stability"[tiab] OR "stretching exercises"[tiab] OR "aerobic exercise"[tiab] OR "mobilization"[tiab] OR "osteopathy"[tiab] OR "neural mobilization"[tiab] OR "ergonomic education"[tiab] OR "posture education"[tiab] OR "transcutaneous electrical nerve stimulation"[tiab] OR "TENS"[tiab] OR "dry needling"[tiab] OR "back school"[tiab] OR "mechanical traction"[tiab] OR "postural training"[tiab]))) | 1943 - 2024 | n = 8,724 |

In Supplementary Table 1 the search strategy performed on 17^th^ of May 2024 is shown below outlining the respective databases, the search terms, publication dates chosen as limiting factors, and number of results from each database are shown.

# **Supplementary Table 2:** Inclusion and exclusion criteria.

| **Inclusion criteria** | **Exclusion criteria** |
| --- | --- |
| - Published in the English language - Peer-reviewed journals - Adults (aged 18 years and older) of any gender with symptomatic lumbar disc herniation, confirmed by MRI or CT scans, representing the primary diagnosis. - Studies where at least 75% of participants meet the above criteria for symptomatic lumbar disc herniation. - Studies comparing conservative (traction, manipulation, exercise) to surgical treatment. - For meta-analysis: Comparison of outcomes in patients treated with varying conservative therapies with comparable outcome measures | - All non-English languages - Commentaries, case reports, narrative reviews, letters to editors, books - Any animal studies and lab-based studies - Studies on children and adolescents (<18 years) - Studies where lumbar disc herniation diagnosis is based solely on myelography. - Populations with co-existing conditions such as inflammatory diseases, significant spinal deformities, or other major spinal pathologies |

In Supplementary Table 2, the inclusion and exclusion criteria used when filtering studies based off search results are shown.

# **Supplementary Table 3:** Table of extracted variables.

| **Extracted variables for qualitative synthesis** | **Extracted variables for quantitative synthesis** |
| --- | --- |
| - Study, Sample size, Study type and design, Population, Intervention, Details of Intervention, Control group, Details of Comparator, Duration of Intervention, Follow-up Period, Outcome Measures, Age (Mean Years ± SD), Gender, Duration of Symptoms, Baseline Severity, Inclusion Criteria, Exclusion Criteria, Frequency of Intervention, Duration of Each Session, Total Duration, | Visual Analog Scale (VAS), VAS at rest pre-treatment, VAS at rest 6 weeks, VAS during movement pre-treatment, VAS during movement 6 weeks, VAS at night baseline, VAS at night 6 weeks, VAS Back Baseline, VAS Back Post-treatment, VAS Back Baseline Morning, VAS Back Baseline Evening, VAS Back 1 week, VAS Back 10 days Morning, VAS Back 10 days Evening, VAS Back 2 weeks, VAS Back 3 weeks, VAS Back 4 weeks, VAS 6 weeks, VAS Back 6 weeks, VAS Back 8 weeks, VAS Back 10 weeks, VAS Back 12 weeks, VAS Back 3 months, VAS Back 6 months, VAS Back 9 months, VAS Back 1 year, VAS Back 2 years, VAS Leg Baseline, VAS Leg Post-treatment, VAS Leg 1 Week, VAS Leg 2 Weeks, VAS Leg 6 Weeks, VAS Leg 8 Weeks, VAS Leg 10 Weeks, VAS Leg 12 Weeks, VAS Leg 4 Weeks, VAS Leg 3 months, VAS Leg 6 months, VAS Leg 9 months, VAS Leg 1 year, VAS Leg 2 years, VAS Leg Post Treatment Control, Visual Analog Scale (VAS) Pain at Rest Pre-test, Visual Analog Scale (VAS) Pain at Rest Post-test, Visual Analog Scale (VAS) General Pain Pre-test, Visual Analog Scale (VAS) General Pain Post-test, Visual Analog Scale (VAS) Pain during Exercise Pre-test, Visual Analog Scale (VAS) Pain during Exercise Post-test, Pain intensity in the lower limb (NPRS) Baseline, Pain intensity in the lower limb (NPRS) 4 sessions, Pain intensity in the lower limb (NPRS) 8 sessions, Pain intensity in the lower limb (NPRS) 2 months, Oswestry Disability Index (ODI), ODI Baseline Treatment, ODI Post-intervention, ODI 10 days Treatment, ODI 2 Weeks Treatment, ODI 3 weeks, ODI 6 weeks, ODI 12 weeks, ODI 1 Month Treatment, ODI 2 months, ODI 10 weeks Treatment, ODI 3 Months Treatment, ODI 6 Months Treatment, ODI 1 Year Treatment, ODI Baseline Control, ODI 5th session Treatment, ODI 1 month Treatment, Oswestry Disability Index Baseline, Oswestry Disability Index Post-treatment, ODI 2 Week, ODI 1 month, ODI 5 weeks, ODI 6 weeks, ODI 10 weeks, ODI 3 months, ODI 6 months, ODI 12 months, SFI Baseline, SFI week 6, SFI week 12, SFI week 26, SFI week 52, SF-36 Baseline, SF-36 week 6, SF-36 week 12, SF-36 week 26, SF-36 week 52, SF-36 Bodily Pain Baseline, SF-36 Bodily Pain 6 weeks, SF-36 Bodily Pain Post-treatment, SF-36 Bodily Pain 3 months, SF-36 Bodily Pain 1 year, SF-36 Physical Function Baseline, SF-36 Physical Function 6 weeks, SF-36 Physical Function 3 months, SF-36 Physical Function 1 year, SF-36 Health status compared with last year Baseline, General Health Mean±SD Baseline, SF-36 Physical Role Difficulty Baseline, SF-36 Physical Role Difficulty 6 weeks, SF-36 Emotional Role Difficulty Baseline, SF-36 Emotional Role Difficulty 6 weeks, SF-36 Vitality Baseline, SF-36 Vitality 6 weeks, SF-36 Mental Component Summary Baseline, SF-36 Mental Component Summary 6 weeks, SF-36 Mental Component Summary 3 months, SF-36 Mental Component Summary 1 year, Short Form-36 (SF-36) Overall, SF-36 baseline, SF-36 2 weeks, SF-36 6 weeks, Short Form-36 (SF-36) Physical Component 12 days, Short Form-36 (SF-36) Mental Component 12 days, Short Form-36 (SF-36) Overall at 6 weeks, Short Form-36 (SF-36) Overall at 8 weeks, Short Form-36 (SF-36) Overall at 6 months, Short Form-36 (SF-36) Overall at 1 year, SF-36 Physical Functioning 6 months, SF-36 Physical Problems 6 months, SF-36 Social Functioning baseline, SF-36 Social Functioning 6 months, SF-46 Energy Vitality 6 months, SF-36 Body Pain 6 months, SF-36 General Health 6 months, SF-36 Health status compared to last year 6 months, SF-36 Mental Health 6 months, Short Form-36 (SF-36) Overall at 2 years, Rolland-Morris Disability Questionnaire (RMDQ), RMDQ baseline, RMDQ post-treatment, RMDQ (0–24) 4 sessions, RMDQ 2 weeks, RMDQ (0–24) 8 sessions, RMDQ after 12 days, RMDQ after 1 month, RMDQ after 6 weeks, RMDQ after 8 weeks, RMDQ after 12 weeks, RMDQ after 2 months, RMDQ after 3 months, RMDQ after 6 months, RMDQ after 9 months, RMDQ after 1 year, RMDQ after 2 years, SBI Baseline, Sciatica Bothersomeness Index (SBI), SBI Post-treatment, SBI 6 weeks, SBI 3 months, SBI 6 months, SBI 9 months, SBI 52 weeks, Neuropathic Symptoms, SLR Baseline, SLR 1 month, Straight Leg Raise (SLR) Test, Straight Leg Raise Degrees Baseline, Straight Leg Raise Degrees 4 sessions, Straight Leg Raise Degrees 8 sessions, Straight Leg Raise Degrees 2 months, PPT Baseline, PPT 2 weeks, PPT 4 weeks, PPTs over the tibial nerve kg/cm² Baseline, PPTs over the tibial nerve kg/cm² 4 sessions, PPTs over the tibial nerve kg/cm² 8 sessions, PPTs over the tibial nerve kg/cm² 2 months, PPTs over the common peroneal nerve kg/cm² Baseline, PPTs over the common peroneal nerve kg/cm² 4 sessions, PPTs over the common peroneal nerve kg/cm² 8 sessions, PPTs over the common peroneal nerve kg/cm² 2 months, DN4 Baseline, DN4 1 month, DN4 3 months, DN4 6 months, LANSS Baseline, LANSS 1 month, LANSS 3 months, LANSS 6 months, Short-form McGill Pain Questionnaire, Short-form McGill Pain Questionnaire Baseline, Short-form McGill Pain Questionnaire 10 days, Short-form McGill Pain Questionnaire 1 month, Short-form McGill Pain Questionnaire 2 months, Short-form McGill Pain Questionnaire 3 months, Short-form McGill Pain Questionnaire 6 months, Passive Knee Extension Test, PKE Baseline, PKE 1 month, PKE 3 months, PKE 6 months, Patient Global Assessment, TUG Baseline, TUG 6 weeks, TUG 12 weeks, TUG 26 weeks, TUG 52 weeks. |

In Supplementary Table 3, a table shows the extracted variables in the qualitative synthesis (systematic review) and quantitative synthesis (meta-analysis).

# **Supplementary Table 4**: Risk of bias analysis of all non-randomised studies (ROBINS-I tool).

| Study | **D1** | **D2** | **D3** | **D4** | **D5** | **D6** | **D7** | **D8** |
| --- | --- | --- | --- | --- | --- | --- | --- | --- |
| Yildirim et al. (2022) | MODERATE | MODERATE | MODERATE | LOW | LOW | MODERATE | MODERATE | MODERATE |
| Thackeray et al. (2017) | MODERATE | MODERATE | MODERATE | MODERATE | MODERATE | LOW | MODERATE | MODERATE |
| Kuligowski et al. (2019) | MODERATE | MODERATE | LOW | LOW | LOW | MODERATE | LOW | MODERATE |
| Asiri et al. (2020) | MODERATE | LOW | LOW | LOW | LOW | LOW | LOW | LOW |
| Shokri et al. (2018) | MODERATE | MODERATE | LOW | LOW | LOW | MODERATE | LOW | MODERATE |
| Weinstein et al. (2006) | MODERATE | SERIOUS | MODERATE | SERIOUS | LOW | MODERATE | MODERATE | CRITICAL |
| Svensson et al. (2014) | MODERATE | LOW | MODERATE | MODERATE | LOW | MODERATE | LOW | MODERATE |
| Ehrler et al. (2016) | LOW | LOW | LOW | LOW | LOW | LOW | LOW | LOW |
| Leemann et al. (2014) | MODERATE | MODERATE | LOW | SERIOUS | MODERATE | SERIOUS | LOW | SERIOUS |
| Annen et al. (2016) | LOW | LOW | MODERATE | MODERATE | LOW | LOW | MODERATE | MODERATE |
| Gugliotta et al. (2016) | MODERATE | MODERATE | LOW | MODERATE | MODERATE | LOW | MODERATE | MODERATE |
| Ghaderi Niri et al. (2024) | MODERATE | MODERATE | LOW | MODERATE | MODERATE | LOW | MODERATE | MODERATE |
| Khanzadeh et al. (2020) | MODERATE | MODERATE | LOW | MODERATE | MODERATE | LOW | MODERATE | MODERATE |
| Tarcău et al. (2022) | MODERATE | MODERATE | LOW | MODERATE | MODERATE | LOW | MODERATE | MODERATE |
| Ye et al. (2015) | MODERATE | MODERATE | LOW | MODERATE | MODERATE | LOW | MODERATE | MODERATE |

Supplementary Table 4 shows the results of the risk of bias analysis of all 15 non-randomised studies using the ROBINS-I tool: Leeman et al. (2014) was scored as serious risk of bias as there was no sufficient analysis of confounding factors as well as selection and measurement bias. Weinstein et al. (2006) was scored as at critical risk of bias, as there were inconsistences between the surgical and nonoperative group.

# **Supplementary Table 5**: Risk of bias analysis of all randomised control trial studies (ROB-2 tool).

| Study | D1 | D2 | D3 | D4 | D5 | D6 |
| --- | --- | --- | --- | --- | --- | --- |
| Taşpınar et al. (2022) | LOW | LOW | LOW | LOW | LOW | LOW |
| Danazumi et al. (2021) | MODERATE | MODERATE | LOW | LOW | LOW | MODERATE |
| Plaza-Manzano et al. (2020) | MODERATE | LOW | LOW | LOW | MODERATE | MODERATE |
| Abdi et al. (2023) | LOW | LOW | LOW | LOW | LOW | LOW |
| Danazumi et al. (2023) | LOW | LOW | LOW | LOW | LOW | LOW |
| Nikoobakht et al. (2016) | LOW | MODERATE | LOW | LOW | LOW | MODERATE |
| Ozturk et al. (2006) | MODERATE | LOW | LOW | LOW | LOW | MODERATE |
| Moustafa et al. (2015) | LOW | LOW | LOW | LOW | LOW | LOW |
| Unlu et al. (2008) | MODERATE | HIGH | LOW | LOW | LOW | HIGH |
| Hahne et al. (2017) | LOW | LOW | LOW | LOW | LOW | LOW |
| Isner-Horobeti et al. (2016) | MODERATE | LOW | LOW | LOW | LOW | MODERATE |
| Kumari et al. (2021) | MODERATE | LOW | LOW | LOW | LOW | MODERATE |
| Choi et al. (2022) | LOW | LOW | LOW | LOW | LOW | LOW |
| He et al. (2006) | LOW | LOW | LOW | LOW | LOW | LOW |
| Salfinger et al. (2015) | LOW | LOW | LOW | LOW | LOW | LOW |
| Peul et al. (2008) | MODERATE | LOW | LOW | LOW | LOW | MODERATE |
| Bello et al. (2019) | HIGH | MODERATE | LOW | LOW | LOW | HIGH |
| Choi et al. (2015) | MODERATE | MODERATE | LOW | LOW | LOW | MODERATE |
| Iosub et al. (2023) | MODERATE | LOW | LOW | LOW | LOW | MODERATE |
| Singh et al. (2022) | LOW | MODERATE | LOW | MODERATE | LOW | MODERATE |
| Lee et al. (2019) | LOW | LOW | LOW | LOW | LOW | LOW |
| França et al. (2019) | LOW | LOW | LOW | LOW | LOW | LOW |
| Muniandy et al. | LOW | LOW | MODERATE | LOW | LOW | MODERATE |
| Murat et al. (2018) | LOW | LOW | LOW | LOW | MODERATE | MODERATE |
| Koçak (2017) | LOW | LOW | LOW | LOW | LOW | LOW |
| Gülşen et al. (2018) | LOW | MODERATE | LOW | LOW | LOW | MODERATE |
| Keles et al. (2017) | LOW | LOW | LOW | LOW | LOW | LOW |
| Luijsterburg et al (2007) | LOW | SOME CONCERNS | LOW | LOW | LOW | LOW |

Supplementary Table 5 shows the results of the risk of bias analysis of all included 28 randomised controlled trials using the Risk of Bias 2 (RoB-2) tool.

# **Supplementary Table 6**: Level of evidence based on the Oxford Centre of Evidence-Based Medicine (OCEBM) Levels of Evidence.

| **Study number** | **Author, Year** | **Level of evidence** |
| --- | --- | --- |
| 1 | Taşpınar et al., (2022) | 1b |
| 2 | Danazumi et al. (2020) | 1b |
| 3 | Plaza-Manzano et al. (2020) | 1b |
| 4 | Yildirim and Gultekin (2022) | 2b |
| 5 | Abdi et al. (2023) | 3b |
| 6 | Danazumi et al. (2023) | 1b |
| 7 | Nikoobakht et al. (2016) | 1b |
| 8 | Thackeray et al., 2017 | 2b |
| 9 | Kuligowski et al., 2019 | 3b |
| 10 | Asiri et al., 2020 | 2b |
| 11 | Shokri et al. (2018) | 1b |
| 12 | Ozturk et al. (2005) | 3b |
| 13 | Moustafa et al. (2012) | 2b |
| 14 | Unlu et al. (2008) | 2b |
| 15 | Hahne et al. (2017) | 1b |
| 16 | Weinstein et al. (2006) | 2b |
| 17 | Svensson et al. (2014) | 2b |
| 18 | Isner-Horobeti et al. (2016) | 2b |
| 19 | Ehrler et al. (2016) | 2b |
| 20 | Kumari et al. (2021) | 1b |
| 21 | Leeman et al. (2014) | 2b |
| 22 | Choi et al. (2022) | 1b |
| 23 | He et al. (2006) | 2b |
| 24 | Annen et al. (2016) | 2b |
| 25 | Gugliotta et al. (2016) | 2b |
| 26 | Salfinger et al. (2014) | 2b |
| 27 | Peul et al (2008) | 1b |
| 28 | Ghaderi Niri et al. (2024) | 2b |
| 29 | Khanzadeh et al (2020) | 2b |
| 30 | Tarcău et al. (2022) | 1b |
| 31 | Ye et al. (2015) | 1b |
| 32 | Bello et al. (2019) | 2b |
| 33 | Choi et al (2015) | 2b |
| 34 | Iosub et al. (2023) | 1b |
| 35 | Singh et al (2022) | 2b |
| 36 | Lee et al. (2019) | 2b |
| 37 | França et al (2019) | 2b |
| 38 | Muniandy et al | 2b |
| 39 | Murat et al (2018) | 2b |
| 40 | Koçak (2017) | 2b |
| 41 | Gülşen et al (2018) | 2b |
| 42 | Keles et al (2017) | 2b |
| 43 | Luijsterburg et al (2007) | 1b |

Supplementary Table 6 shows the results of the risk of bias analysis of all included studies using the Oxford Centre of Evidence-Based Medicine (OCEBM) Levels of Evidence tool.

# **Supplementary Table 7**: GRADE (Grading of Recommendations, Assessment, Development and Evaluations) scoring for all studies.

| Study | GRADE Risk of Bias | GRADE Imprecision | GRADE Inconsistency | GRADE Indirectness | Publication bias | GRADE Overall |
| --- | --- | --- | --- | --- | --- | --- |
| Taşpınar et al., 2022 | MODERATE | LOW | LOW | MODERATE | LOW | MODERATE |
| Danazumi et al. (2020) | MODERATE | LOW | LOW | MODERATE | LOW | MODERATE |
| Yildirim and Gultekin (2022) | MODERATE | LOW | LOW | LOW | LOW | MODERATE |
| Abdi et al. (2023) | MODERATE | MODERATE | LOW | LOW | LOW | MODERATE |
| Danazumi et al. (2023) | MODERATE | MODERATE | LOW | LOW | LOW | MODERATE |
| Nikoobakht et al. (2016) | MODERATE | MODERATE | LOW | LOW | LOW | MODERATE |
| Thackeray et al., 2017 | MODERATE | HIGH | HIGH | MODERATE | LOW | LOW |
| Kuligowski et al., 2019 | MODERATE | HIGH | LOW | LOW | HIGH | MODERATE |
| Asiri et al., 2020 | MODERATE | MODERATE | LOW | MODERATE | LOW | MODERATE |
| Shokri et al. (2018) | MODERATE | HIGH | LOW | MODERATE | LOW | LOW |
| Ozturk et al. (2005) | MODERATE | MODERATE | LOW | LOW | LOW | MODERATE |
| Moustafa et al. (2012) | MODERATE | MODERATE | LOW | LOW | LOW | MODERATE |
| Unlu et al. (2008) | MODERATE | MODERATE | LOW | LOW | LOW | MODERATE |
| Hahne et al. (2017) | MODERATE | HIGH | LOW | LOW | LOW | MODERATE |
| Weinstein et al. (2006) | MODERATE | LOW | LOW | LOW | LOW | MODERATE |
| Svensson et al. (2014) | MODERATE | MODERATE | LOW | LOW | MODERATE | MODERATE |
| Isner-Horobeti et al. (2016) | MODERATE | HIGH | LOW | LOW | MODERATE | MODERATE |
| Ehrler et al. (2016) | MODERATE | MODERATE | MODERATE | LOW | LOW | MODERATE |
| Kumari et al. (2021) | MODERATE | MODERATE | MODERATE | LOW | LOW | MODERATE |
| Leeman et al. (2014) | MODERATE | LOW | LOW | LOW | LOW | MODERATE |
| Choi et al. (2022) | MODERATE | MODERATE | LOW | LOW | LOW | MODERATE |
| He et al. (2006) | HIGH | MODERATE | LOW | MODERATE | LOW | LOW |
| Annen et al. (2016) | MODERATE | MODERATE | LOW | MODERATE | LOW | MODERATE |
| Gugliotta et al. (2016) | MODERATE | MODERATE | LOW | LOW | LOW | MODERATE |
| Salfinger et al. (2014) | MODERATE | HIGH | MODERATE | LOW | LOW | MODERATE |
| Peul et al (2008) | LOW | LOW | LOW | LOW | LOW | HIGH |
| Ghaderi Niri et al. (2024) | LOW | LOW | LOW | MODERATE | LOW | MODERATE |
| Khanzadeh et al (2020) | MODERATE | MODERATE | MODERATE | MODERATE | LOW | MODERATE |
| Ye et al. (2015) | MODERATE | MODERATE | MODERATE | MODERATE | LOW | MODERATE |
| Bello et al. (2019) | MODERATE | MODERATE | LOW | MODERATE | LOW | MODERATE |
| Choi et al (2015) | LOW | MODERATE | LOW | MODERATE | LOW | MODERATE |
| Muniandy. et al (2019) | MODERATE | LOW | MODERATE | HIGH | LOW | MODERATE |
| Iosub et al. (2023) | MODERATE | LOW | MODERATE | HIGH | LOW | MODERATE |
| Singh et al (2022) | MODERATE | MODERATE | LOW | LOW | LOW | MODERATE |
| Lee et al. (2019) | MODERATE | LOW | MODERATE | HIGH | MODERATE | MODERATE |
| França et al (2019) | MODERATE | MODERATE | LOW | LOW | LOW | MODERATE |
| Murat et al (2018) | MODERATE | MODERATE | LOW | LOW | LOW | MODERATE |
| Koçak, 2017 | MODERATE | MODERATE | LOW | LOW | MODERATE | MODERATE |
| Gülşen et al (2018) | LOW | MODERATE | LOW | LOW | MODERATE | MODERATE |
| Keles et al (2017) | MODERATE | MODERATE | LOW | LOW | MODERATE | MODERATE |
| Luijsterberg (2007) | MODERATE | HIGH | MODERATE | LOW | HIGH | MODERATE |

Supplementary Table 7 shows the results of the risk of bias analysis of all included studies using the GRADE (Grading of Recommendations, Assessment, Development and Evaluations) scoring.
